# Handedness is related to neural mechanisms underlying hemispheric lateralization of face processing

**DOI:** 10.1038/srep27153

**Published:** 2016-06-02

**Authors:** Stefan Frässle, Sören Krach, Frieder Michel Paulus, Andreas Jansen

**Affiliations:** 1 Translational Neuromodeling Unit (TNU), Institute for Biomedical Engineering, University of Zurich & ETH Zurich, CH-8032 Zurich, Switzerland; 2 Laboratory for Multimodal Neuroimaging (LMN), Department of Psychiatry, University of Marburg, D-35039 Marburg, Germany; 3 Department of Child- and Adolescent Psychiatry, University of Marburg, D-35039 Marburg, Germany; 4 Social Neuroscience Lab | SNL, Department of Psychiatry and Psychotherapy, University of Lübeck, D-23538 Lübeck, Germany; 5 Core Facility Brainimaging, Department of Psychiatry, University of Marburg, D-35039 Marburg, Germany

## Abstract

While the right-hemispheric lateralization of the face perception network is well established, recent evidence suggests that handedness affects the cerebral lateralization of face processing at the hierarchical level of the fusiform face area (FFA). However, the neural mechanisms underlying differential hemispheric lateralization of face perception in right- and left-handers are largely unknown. Using dynamic causal modeling (DCM) for fMRI, we aimed to unravel the putative processes that mediate handedness-related differences by investigating the effective connectivity in the bilateral core face perception network. Our results reveal an enhanced recruitment of the left FFA in left-handers compared to right-handers, as evidenced by more pronounced face-specific modulatory influences on both intra- and interhemispheric connections. As structural and physiological correlates of handedness-related differences in face processing, right- and left-handers varied with regard to their gray matter volume in the left fusiform gyrus and their pupil responses to face stimuli. Overall, these results describe how handedness is related to the lateralization of the core face perception network, and point to different neural mechanisms underlying face processing in right- and left-handers. In a wider context, this demonstrates the entanglement of structurally and functionally remote brain networks, suggesting a broader underlying process regulating brain lateralization.

Faces convey a wealth of information, such as a person’s identity, emotions, intentions, attractiveness, or trustworthiness^1^ – aspects crucial for social interactions in everyday life. The neural network that underlies the processing of faces has been found to be distributed across multiple brain regions, but mainly lateralized to the right hemisphere^2^. Specifically, in prosopagnosia, brain lesions lateralized to the right hemisphere are sufficient to induce impairments in face recognition^3^,^4^. Furthermore, with help of modern neuroimaging techniques like functional magnetic resonance imaging (fMRI), supporting evidence for the right-hemispheric lateralization of the face perception network has been provided^5^,^6^,^7^. As such, the functional asymmetry of the face perception network is often referred to as a fundamental lateralization of the human brain^8^. However, as these observations rest on studies which tested healthy subjects or prosopagnostic patients who were right-handed, results may not generalize to the whole population. This is critical as handedness relates to the topology of neural networks underlying various cognitive functions^8^. Most prominently, left-handers have more bilateral language processing^9^,^10^,^11^. For instance, Knecht and colleagues demonstrated that only a small proportion of right-handers (4%) exhibit right-hemispheric dominance of the language network, whereas this share increases to at least 27% in left-handers. Similarly, differences in hemispheric lateralization related to handedness have also been observed for spatial attention^12^. In particular, the authors demonstrated bilateral activation during a spatial attention task for those subjects who displayed right-hemispheric dominance for language. This suggests that hemispheric lateralization for language and spatial attention interfere with one another to some extent and yield a hemispheric shift by recruitment of the opposite hemisphere. Furthermore, handedness-related differences have also been revealed for the hemispheric lateralization of the motor system^13^,^14^. Consistent with such a close link between handedness and lateralization, genetic models^15^ have provided a description for both handedness and brain asymmetry, thus suggesting a shared molecular basis mediating the observed associations^16^.

Extending these observations, two more recent neuroimaging studies have found a relationship between handedness and the hemispheric lateralization of face processing. Willems *et al.* demonstrated that the lateralization of the fusiform face area (FFA)^6^ depends on handedness: While the FFA was lateralized to the right hemisphere in right-handers, such asymmetric hemispheric lateralization was absent in left-handers^17^. Notably, the authors focused on the FFA, while neglecting another, equally important component of the core face perception network: the occipital face area (OFA)^5^. Another study took this into account by investigating the relationship between handedness and the lateralization of both OFA and FFA^18^. Besides replicating the above-mentioned finding with respect to the FFA, the authors reported that the OFA was right-lateralized in both right- and left-handers, with no difference in the degree of lateralization. The authors concluded that handedness relates to hemispheric lateralization of the face perception network only at the hierarchical level of the FFA.

While these findings have contributed to our understanding of the variability in hemispheric lateralization in the human brain, the putative processes by which handedness and brain asymmetry are coupled remain vague. To overcome this limitation, generative models provide a promising avenue as they allow us to pinpoint handedness-related effects on the effective (i.e., directed) connectivity among brain regions. Here, we propose such a framework to address the relation of handedness and the lateralization of the face perception network described earlier. Specifically, we aim to test whether more bilateral activation of the FFA in left-handers is attributable to an enhanced recruitment of the left FFA during face processing, or conversely, whether it is due to a less pronounced role of the right FFA (note that a similar notion has also been addressed in the context of language processing^19^). Furthermore, from neuroimaging studies that test measures of functional activation, it remains unknown whether differences in the recruitment of bilateral FFA in left-handers manifest in the intra- or interhemispheric integration of the network, or both, and whether the observed differences arise from influences at early or late stages within the processing hierarchy of the core face perception network. Delineating these mechanisms yields deeper insights into the lateralized processes in the face perception network, and could also advance our understanding of the pathophysiology of diseases in which face processing is impaired (e.g., prosopagnosia).

In this line, effective connectivity studies on the generative processes underlying face processing in the human brain have recently emerged, and have provided valuable insights^20^,^21^,^22^,^23^. However, these studies have merely focused on the intrahemispheric connections, while neglecting the interhemispheric connections among face-sensitive regions. Such an approach, however, might leave the above-mentioned questions on how handedness relates to the mechanisms underlying hemispheric lateralization of the face perception network unanswered. To this end, we have recently introduced a novel approach to investigate the effective connectivity among face-sensitive regions in a bilateral network, taking into account intra- *and* interhemispheric integration^24^. Using dynamic causal modeling (DCM)^25^ for fMRI, we emphasized the relevance of interhemispheric integration during face processing and provided a mechanistic description of the right-hemispheric lateralization of the core face perception network^24^. Further analyses demonstrated the utility of this framework by showing high test-retest reliability for both conventional brain activation and effective connectivity measures^26^. Overall, this approach provides a promising avenue to probe the effects of handedness on the effective connectivity within the face perception network.

In the present study, we tested 20 left- and 20 (matched) right-handed subjects by presenting either faces, objects or scrambled images in the left or right visual field, while subjects fixated a cross in the center of a screen^24^,^26^. Differences in the effective connectivity patterns of right- and left-handers were assessed using DCM. Furthermore, we asked whether differences in the functional organization of the core face perception network also manifest in other modalities, specifically in the gray matter volume and in physiological measures of cognitive processing (i.e., pupil dynamics). These two properties were selected because of recent evidence from our group that suggested an informative link of hemispheric lateralization, in particular the interhemispheric integration, with gray matter volume and pupil dynamics during face processing^24^. Hence, if handedness relates to differences in the organization of the core face perception network, one might also expect similar effects on these variables. We therefore aimed at testing for handedness-related differences in gray matter volume and pupil dynamics in an exploratory manner. Implementing such a multimodal approach, combing DCM, structural MRI and pupillometry, we attempted to more closely delineate the nature of differential neural mechanisms underlying face processing in right- and left-handers.

## Results

**Figure 1:**
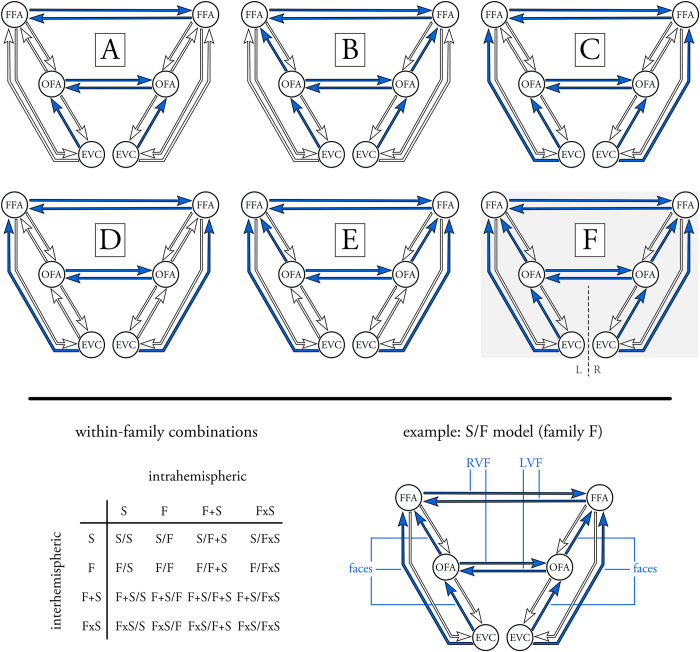
Model space implementing a systematic variation of different hypotheses on the effective connectivity in the face perception network. Notably, all models were identical with regard to their endogenous connectivity and driving inputs. Driving inputs (not shown here) were set to allow for effects of the visual stimuli on the neural activity in the contralateral EVC (i.e., RVF modulated left EVC, LVF modulated right EVC). Within each hemisphere, reciprocal endogenous connections were set between all three regions of the network (i.e., EVC, OFA and FFA). Additionally, reciprocal interhemispheric connections were set between bilateral OFA, and between bilateral FFA, but not between EVC. Arrows indicate the presence and directionality of the endogenous connections. Models differed with respect to their modulatory connectivity pattern and were divided into 6 different model families according to the site where experimental inputs perturbed the intrahemispheric forward connections (*top*). Within each family, models differed with regard to the type of modulatory influence on the intra- and interhemispheric connections (*bottom, left*). Each connection was either modulated by 1) the visual field (S), 2) the perception of faces (F), 3) both the perception of faces and the visual field (F + S), or 4) the perception of faces conditional on the visual field (F × S). All four possibilities exist for intra- and interhemispheric connections. Models are named by first listing the type of interhemispheric modulation, followed by the type of intrahemispheric modulation. Example of the S/F model of Family F where all forward intrahemispheric connections were modulated by the processing of faces and the interhemispheric connections were modulated by the visual field (*bottom, right*). EVC = early visual cortex; FFA = fusiform face area; OFA = occipital face area; LVF = left visual field; RVF = right visual field; L = left hemisphere; R = right hemisphere.

We adopted a recent effective connectivity approach to investigate how handedness relates to hemispheric lateralization during face processing. First, we assessed handedness-related differences in the hemispheric lateralization of face-sensitive activation in the core network, replicating earlier fMRI studies. Second, we aimed to provide a mechanistic description of these observations by testing for differential effective connectivity between right- and left-handers using DCM and a comprehensive model space (Fig. 1). Third, we asked whether differences in the functional organization also manifest in measures of other modalities, specifically brain anatomy and pupillometry.

### Handedness relates to hemispheric lateralization of face-sensitive BOLD activation

#### Brain activity during face perception

**Figure 2:**
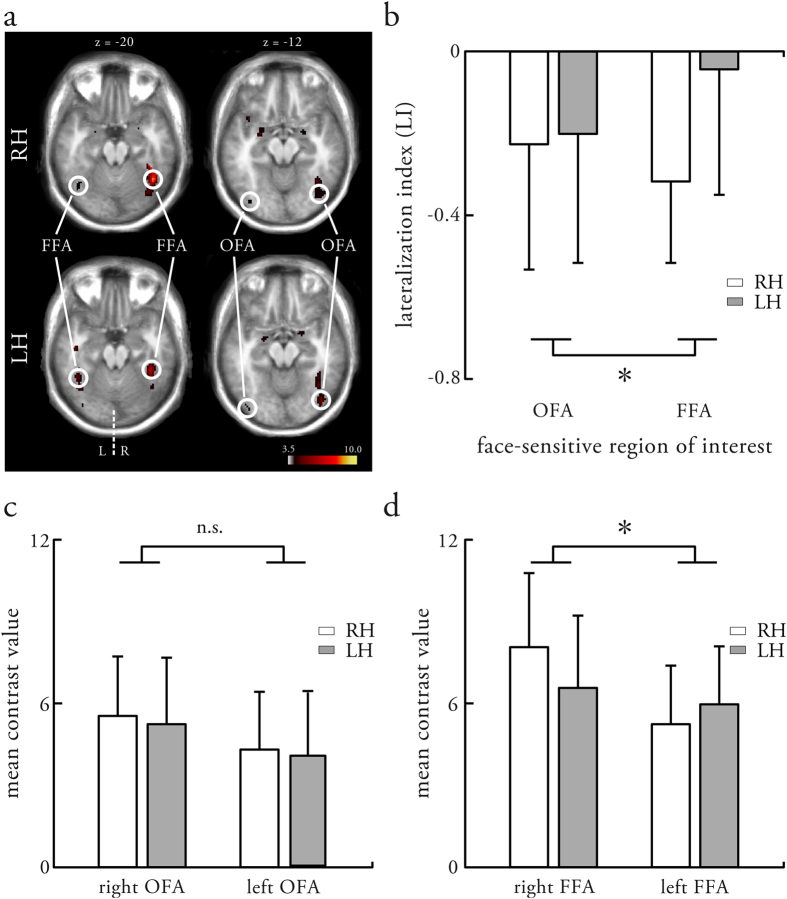
Cerebral lateralization of the core face perception network in right- and left-handers. (**a**) BOLD activation pattern shows brain regions that were more activated during the perception of faces as compared to objects and scrambled images (differential contrast: [2*F]-[O + S]) for right-handers (*top*) and left-handers (*bottom*). Activation patterns are thresholded at a liberal voxel-level threshold of *p* < 0.001 (uncorrected) and displayed on an anatomical template image. For displaying purposes, activation in the cerebellum was excluded using an exclusive anatomical mask. FFA = fusiform face area; OFA = occipital face area; LH = left-handers; RH = right-handers; L = left hemisphere; R = right hemisphere. (**b**) Mean and standard deviation of the lateralization indices (LI) of BOLD activity in OFA and FFA for right-handers (white) and left-handers (gray). The * denotes a significant handedness × region interaction, suggesting that right- and left-handers showed differential hemispheric lateralization of BOLD activity in the FFA, but not in the OFA. (**c**) Mean and standard deviation of the mean contrast value in the OFA for right-handers (white) and left-handers (gray). The n.s. illustrates that there was no significant handedness × hemisphere interaction, suggesting that right- and left-handers showed similar hemispheric lateralization of BOLD activity in the OFA. (**d**) Mean and standard deviation of the mean contrast value in the FFA for right-handers (white) and left-handers (gray). The * denotes a significant handedness × hemisphere interaction, suggesting that right- and left-handers showed differential hemispheric lateralization of BOLD activity in the FFA.

Using separate random effects group analyses (one-sample *t*-tests) for right- and left-handers, we identified brain regions that responded more strongly to faces than to objects and scrambled images (*p* < 0.001, uncorrected). For both handedness groups, we similarly found activation in the inferior occipital gyrus and the fusiform gyrus in both hemispheres, referring to bilateral OFA and FFA, respectively (Fig. 2a). Additional face-sensitive BOLD activations were observed (Table 1), but were more diverse between right- and left-handers and were thus not considered in subsequent fMRI and DCM analyses. This is also consistent with the approach from previous activation^18^ and effective connectivity studies^24^,^26^, which focused on the hemispheric lateralization in the core face perception network.

#### Handedness-related differences in the hemispheric lateralization of face-sensitive activation in OFA and FFA

Following previous work^18^, we aimed to test handedness-related differences in the hemispheric lateralization of face-sensitive BOLD activation in the core face perception network. For each subject, we therefore computed an LI for OFA and FFA, separately. Using a 2 × 2 mixed effects ANOVA (between-subject factor: handedness, within-subject factor: region), we found a significant handedness × region interaction (*F*_(1,38)_ = 4.36, *p* = 0.04; Fig. 2b). Post-hoc two-sample *t*-tests revealed that face-sensitive activation was significantly more strongly lateralized to the right hemisphere in right-handers for the FFA (*t*_(38)_ = −3.31, *p* < 0.01), but not for the OFA (*t*_(38)_ = −0.23, *p* = 0.82). In fact, for left-handers, no significant lateralization could be observed for the FFA (mean and std: LI = −0.04 ± 0.31; *t*_(19)_ = −0.63, *p* = 0.54). These results replicate earlier findings on how hemispheric lateralization of face-sensitive activation patterns differs with regard to handedness^17^,^18^.

To delineate these handedness-related differences in the hemispheric lateralization of face-sensitive activation in the OFA and FFA, we investigated the univariate responses of the four face-sensitive regions in subsequent post-hoc analyses. To this end, we estimated the mean contrast value for the [2*F]-[O + S] contrast in each of the subject-specific ROIs. Using two separate 2 × 2 mixed effects ANOVA (between-subject factor: handedness, within-subject factor: hemisphere), we found no significant handedness × hemisphere interaction for the OFA (*F*_(1,38)_ = 0.02, *p* = 0.88; Fig. 2c). Post-hoc two-sample *t*-tests confirmed that there were no handedness-related differences in face-sensitive activation, neither for the left OFA (*t*_(38)_ = 0.26, *p* = 0.80) nor the right OFA (*t*_(38)_ = 0.39, *p* = 0.70). Conversely, a significant handedness × hemisphere interaction was observed for the FFA (*F*_(1,38)_ = 9.50, *p* < 0.01; Fig. 2d). However, post-hoc two-sample *t*-tests did not reveal handedness-related differences for the left FFA (*t*_(38)_ = −1.03, *p* = 0.31) or the right FFA (*t*_(38)_ = 1.71, *p* = 0.10). Hence, from these analyses, it remains unclear as to whether handedness-related differences in hemispheric lateralization during face perception are due to a relative enhancement of the left FFA or a reduction of the right FFA in left-handers.

#### Specificity of handedness-related differences in hemispheric lateralization

To provide a more comprehensive analysis of the observed handedness-related differences in the hemispheric lateralization of BOLD activation, we asked two questions: First, are handedness-related differences in the hemispheric lateralization of OFA and FFA restricted to face processing or can similar effects be observed for the visual field baseline contrasts (i.e., LVF, RVF) as well? Second, are handedness-related differences restricted to the face-sensitive representations (i.e., core face perception network) or do these effects represent an overall lateralization change in object perception (i.e., in object-sensitive cortex)? These analyses suggested that handedness-related differences in hemispheric lateralization are restricted to face-sensitive activations in the core face perception network (Supplementary Note S1, Supplementary Fig. S1).

### Handedness relates to the effective connectivity in the face perception network

#### Bayesian model selection

In a next step, we analyzed the effective connectivity among the face-sensitive brain regions by means of DCM. For each subject, we computed 96 models, which were grouped into six different model families (family A-F, see Methods for detailed information). Random effects Bayesian model selection (BMS) at the family level^27^ was used to compare models based on their negative free energy. We found that the random effects BMS procedure selected different winning families in right- and left-handers. For right-handers, family B was the most likely family (expected probability: 0.50; exceedance probability: 0.81), suggesting that only the intrahemispheric forward connections from EVC to OFA, and from OFA to FFA, were modulated by the experimental manipulations. For left-handers, the most likely family was family F (expected probability: 0.72; exceedance probability: 1.00), indicating that all intrahemispheric forward connections were modulated. This points to a more decisive role of the modulatory influences on the connections from EVC to FFA in left-handers.

The above-mentioned procedure for assessing group effects at the level of random-effects BMS has been utilized by the vast majority of studies so far (cf.^28^). Critically, however, this approach is limited because it does not explicitly account for the possibility that the same family describes both groups. In an additional analysis, we therefore asked whether there is evidence for different winning families between the two handedness groups (rather than a difference in evidence). To this end, we performed a between-group comparison of model frequencies^28^. This analysis did not support the claim of handedness-related differences in the model frequencies (i.e., posterior probability of model frequencies being equal for right- and left-handers: *p* > 0.05). Hence, although different winning families were selected by random effects BMS at the family level, these (between-group) differences were not significant. In a next step, we thus aimed at investigating the relation between handedness and effective connectivity of the face perception network at a more fine-grained level by looking at the level of model parameters.

#### Endogenous parameter and driving input estimates

**Figure 3:**
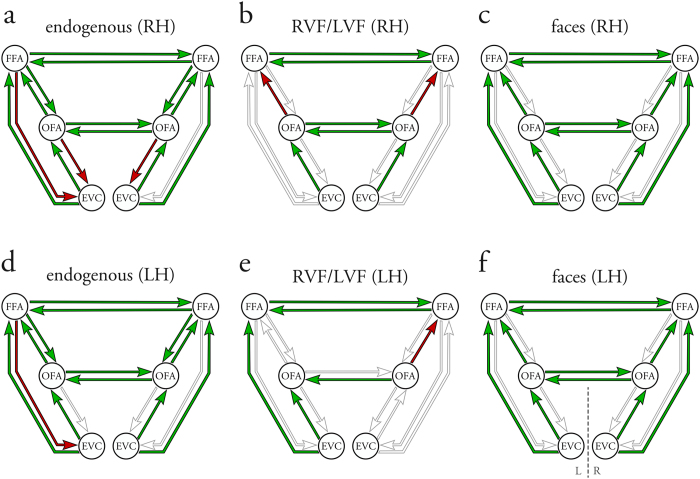
Effective connectivity pattern of the bilateral face perception network in right-handers (*top*) and left-handers (*bottom*). (**a**,**d**) Endogenous connectivity (A-matrix) among the visual input regions (EVC) and the core regions of the face perception network (OFA, FFA) for right-handers (**a**) and left-handers (**d**). Green arrows indicate excitatory connections and red arrows indicate inhibitory connections (*p* < 0.05, FDR-corrected). Gray arrows indicate that the respective endogenous connection did not reach significance at the above-mentioned statistical threshold. (**b**,**e**) Modulatory influences (B-matrix) on the endogenous connections when perceiving any stimulus conditional on the visual field for right-handers (**b**) and left-handers (**e**). (**c**,**f**) Modulatory influences on the connectivity when perceiving faces regardless of the visual field for right-handers (**c**) and left-handers (**f**). Green arrows indicate excitatory modulatory influences and red arrows indicate inhibitory modulatory influences (*p* < 0.05, FDR-corrected). Gray arrows indicate endogenous connections that were not modulated by the experimental manipulations, either because no modulatory influences were define (feedback connections) or because the modulatory influences did not reach significance at the above-mentioned statistical threshold. For a full description (including the actual values of the connection strengths) of the endogenous and modulatory parameter estimates, see Tables 2 and 3, respectively. LVF = left visual field; RVF = right visual field; LH = left-handers; RH = right-handers; L = left hemisphere; R = right hemisphere.

Individual connectivity parameters were calculated using Bayesian model averaging (BMA)^27^ across all 96 models (i.e., across the whole model space) within the standard Occam’s window (including only models with a posterior odds ratio above *p* > 0.05; see Methods for detailed information). Group-level results were then estimated for right- and left-handers separately, by entering the individual parameter estimates into summary statistics (one-sample *t*-tests, FDR-corrected for multiple comparisons). For right- and left-handers, we found that the intrahemispheric forward connections among the three brain regions, as well as the feedback connection from FFA to OFA, were excitatory in both hemispheres (Fig. 3a–d and Table 2). Additionally, all feedback connections to EVC were inhibitory. However, this was not significant for the connection from right FFA to EVC in right-handers, and was only significant for the connection from left FFA to EVC in left-handers. Furthermore, right- and left-handers displayed excitatory reciprocal interhemispheric connections among the homotopic face-sensitive regions.

Driving input estimates indicated that stimuli in either visual field positively modulated neural activity in contralateral EVC for both right- (right EVC: 0.794 ± 0.537 Hz; *t*_(19)_ = 9.18, *p* < 0.001; left EVC: 0.895 ± 0.450 Hz; *t*_(19)_ = 8.90, *p* < 0.001) and left-handers (right EVC: 0.663 ± 0.278 Hz; *t*_(19)_ = 10.66, *p* < 0.001; left EVC: 0.731 ± 0.334 Hz; *t*_(19)_ = 9.78, *p* < 0.001).

#### Handedness-related differences in endogenous connectivity and driving inputs

We then tested for differential endogenous connectivity between right- and left-handers by means of a two-sample *t*-test for each parameter (Table 2). However, endogenous connection strengths were not significantly different between the two handedness groups (all *p* > 0.05, FDR-corrected). Similarly, right- and left-handers did not significantly differ with regard to their driving input estimates. This suggests that there were no task-independent differences in the effective connectivity patterns between the two groups.

#### Modulatory parameter estimates

Adding to the endogenous connectivity, intra- and interhemispheric connections were modulated both by the visual field of the stimulus presentation (regardless of the stimulus category) and the processing of faces. For right-handers, stimuli that were shown in the RVF positively modulated the forward connections from left EVC to left OFA and negatively modulated the connections from left OFA to left FFA (Fig. 3b and Table 3). Additionally, RVF positively modulated the interhemispheric connections from the left face-sensitive areas to their right homotopic counterparts. Stimuli that were presented in the LVF evoked the exact mirror-inverted modulatory connectivity pattern. For left-handers, the same overall pattern of modulatory influences by RVF/LVF was observed, although some of the parameters did not reach significance (Fig. 3e and Table 3). Additionally, the intrahemispheric connection from left EVC to left FFA was positively modulated by RVF for left-handers, but not for right-handers.

The presentation of faces positively modulated the intrahemispheric forward connections among the three ROIs in each hemisphere (Fig. 3c–f and Table 3). This suggests that faces are processed in a hierarchical manner via OFA to FFA, but can also surpass the OFA via connections from EVC to FFA. Notably, such a connection from EVC to FFA might represent either a direct connection or an indirect route via other brain regions not explicitly captured in our model. Furthermore, the interhemispheric connections among the homotopic face-sensitive regions were positively modulated by the faces, suggesting a crucial role of interhemispheric recruitment during face processing^24^.

#### Handedness-related differences in modulatory connectivity

**Figure 4:**
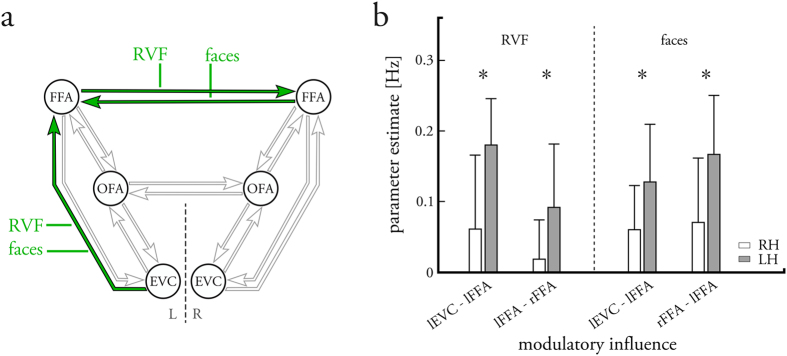
Effects of handedness on the modulatory connectivity of the bilateral core face perception network. (**a**) Differential modulatory connectivity (B-matrix) where green arrows indicate significantly stronger modulatory influences in left- as opposed to right-handers (*p* < 0.05, FDR-corrected). Gray arrows indicate endogenous connections that did not show differential modulatory connectivity, either because no modulatory influences were define (feedback connections) or because the modulatory influences were not significantly different between right- and left-handers at the above-mentioned statistical threshold. (**b**) Strength of the modulatory parameter estimates that differed significantly in right-handers (white) as compared to left-handers (gray) for the two modulatory influences by the right visual field (*left of the dashed line*) and the two modulatory influences by face processing (*right of the dashed line*). Bars indicate the mean parameter estimates and error bars the respective standard deviations. For a full description of the handedness-related differences in the endogenous and modulatory parameter estimates, see Tables 2 and 3, respectively. RVF = right visual field; LH = left-handers; RH = right-handers; L = left hemisphere; R = right hemisphere.

Finally, we tested for differences in the modulatory connectivity patterns between right- and left-handers. We found significant differences between right- and left-handers for the modulatory influences by RVF, but not for those by LVF (Fig. 4 and Table 3). Specifically, the intrahemispheric forward connection from left EVC to left FFA was significantly more strongly modulated in left-handers compared to right-handers (*p* < 0.05, FDR-corrected). Similarly, left-handers showed stronger modulations on the interhemispheric connection from left to right FFA by RVF. Furthermore, we observed significant differences related to handedness for the face-specific modulatory influences: For left-handers, the intrahemispheric forward connection from left EVC to left FFA, and the interhemispheric connection from right to left FFA were significantly more strongly modulated by face processing. Conversely, no significantly larger modulatory influences were observed in right-handers compared with left-handers. Overall, these results suggest that right- and left-handers show differential modulatory connectivity for connections from or towards the left FFA, whereas they do not differ significantly regarding the modulatory influences for connections from or towards bilateral OFA.

As suggested by one of our reviewers, the face-sensitive contrast utilized in the present study ([2*F]-[O + S]) is not the most appropriate contrast for identifying face-specificity. We therefore repeated the analyses on how handedness relates to the hemispheric lateralization of face-sensitive BOLD activation and to the effective connectivity in the face perception network when defining bilateral OFA and FFA based on the conjunction analysis ([F-O] ∩ [F-S]). Notably, three right-handers and three left-handers had to be excluded from the analyses as they did not show consistent activation in all four ROIs for the statistical threshold (*p* < 0.05, uncorrected). For the remaining subjects, we found virtually the same results when selecting ROIs based on the face-selective conjunction as compared to our initial analysis (Supplementary Note S2, Supplementary Fig. S2, and Supplementary Tables S1 and S2).

### Handedness relates to brain anatomy and physiological markers of face perception

#### Handedness-related differences in brain anatomy

**Figure 5:**
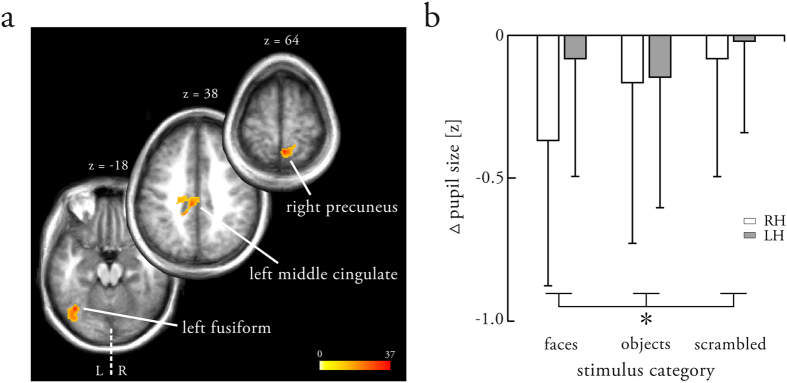
Effects of handedness on structural and physiological markers of face processing. (**a**) Clusters show brain regions with larger gray matter volume in left- as opposed to right-handers at a cluster-extent threshold of *p* < 0.05, FWE-corrected (voxel level threshold of *p* < 0.001, uncorrected). Patterns were displayed on an anatomical template image. (**b**) Changes in pupil size across face, object and scrambled blocks for right-handers (white) and left-handers (gray). For each trial, changes in pupil size were quantified by a single number representing the difference between the average pupil sizes in a time interval between 9 s to 11 s and during the initial 50 ms (see Methods for detailed information). The * denotes that there was a significant handedness × stimulus interaction. Bars indicate the mean change in pupil size and error bars the respective standard deviations. LH = left-handers; RH = right-handers.

Given the observed effects of handedness at the level of BOLD activation and effective connectivity, we asked whether handedness-related differences also manifest in structural properties (i.e., brain anatomy) of the face perception network. We therefore tested whether gray matter volume, as revealed by VBM, differed between right- and left-handers. A random effects group analysis (between-subject ANOVA) revealed handedness-related differences in gray matter volume in three clusters (Fig. 5a) at a cluster extent threshold of *p* < 0.05, FWE-corrected (voxel-level threshold of *p* < 0.001, uncorrected). These clusters were located in the right precuneus (k_E_ = 388; MNI: 3, −52, 64), the left middle cingulate (k_E_ = 405; MNI: −8, −30, 38), and most prominently the left fusiform gyrus (k_E_ = 358; MNI: −39, −68, −18). In fact, the VBM cluster in the left fusiform gyrus partly overlapped with the group-level face-sensitive BOLD activations in the left fusiform gyrus for both right- and left-handers (Supplementary Fig. S3). Furthermore, an additional cluster (voxel level threshold of *p* < 0.001, uncorrected) was observed bilaterally in the supplementary motor area (k_E_ = 212; MNI: −4, 16, 56), although this cluster did not survive a proper cluster extent threshold correction. Post-hoc two-sample *t*-tests suggested that all clusters displayed larger gray matter volume in left- as opposed to right-handers. Conversely, no clusters indicated a larger gray matter volume in right- as compared to left-handers (even at the more liberal statistical threshold).

#### Handedness-related differences in pupil dynamics

So far, we have provided evidence indicating that right- and left-handers exhibit different neural mechanisms during face processing, as illustrated by differences in the hemispheric lateralization of face-sensitive activation in the FFA, modulatory connectivity, and brain anatomy. This begs the question of whether these differences manifest not only in structural and functional brain measures, but also in physiological markers of face processing. To this end, we tested in an exploratory manner for handedness-related differences in pupil dynamics (representing slow changes in pupil size over the blocks; see Methods for detailed information). A 3-way repeated measures ANOVA (between-subject factor: handedness, within-subject factor: stimulus, hemifield) revealed a significant main effect of stimulus (*F*_(2,37)_ = 5.28, *p* = 0.01) and a significant handedness × stimulus interaction (denoted by the * in Fig. 5b; *F*_(2,37)_ = 4.20, *p* = 0.02). We did not observe a significant main effect of handedness (*F*_(2,37)_ = 0.94, *p* = 0.34) or hemifield (*F*_(2,38)_ = 3.11, *p* = 0.09). Moreover, there was no significant handedness × hemifield (*F*_(2,38)_ = 0.20, *p* = 0.66), stimulus × hemifield (*F*_(2,37)_ = 1.07, *p* = 0.35), or handedness × stimulus × hemifield interaction (*F*_(2,37)_ = 1.81, *p* = 0.18). Post-hoc analyses were conducted to investigate the significant effects more closely. First, to better understand the main effect of stimulus, we performed pairwise comparisons of the pupil dynamics for the different stimulus categories (regardless of handedness or hemifield) using post-hoc one-sample *t*-tests. These analyses showed that pupil dynamics differed between faces and scrambled images (*t*_(39)_ = −3.08, *p* = 0.004), whereas no significant differences were observed between faces and objects (*t*_(39)_ = −1.13, *p* = 0.26), or between objects and scrambled images (*t*_(39)_ = −1.46, *p* = 0.15). Second, to delineate the nature of the handedness × stimulus interaction, we conducted post-hoc two-sample *t*-tests for each stimulus category separately. These tests revealed that the change in pupil size over face blocks differed between right- and left-handers at trend level (*t*_(38)_ = −1.92, *p* = 0.06), with a stronger decrease in pupil size in right-handers, whereas no differences were observed between the handedness groups for objects (*t*_(38)_ = −0.12, *p* = 0.91) or scrambled images (*t*_(38)_ = −0.52, *p* = 0.61). This suggests that right- and left-handers processed faces differently, whereas objects and scrambled images evoked similar responses.

## Discussion

In the present study, we examined the neural mechanisms underlying face processing in right- and left-handers. First, our data replicated previous findings^17^,^18^ by demonstrating that handedness was related to the lateralization of BOLD activation only at the hierarchical level of the FFA but not the OFA. Second, we extended current views by providing a mechanistic description of handedness-related differences in the functional dynamics of the core face perception network. These analyses revealed an enhanced face-specific intra- and interhemispheric recruitment of the left FFA in left-handers. Third, these observations were corroborated by differences in brain anatomy and physiological markers of cognitive processes (i.e., pupil dynamics). Specifically, gray matter volume in the left fusiform gyrus was increased in left-handers, and pupil dynamics differed between the two groups only during face perception. Collectively, DCM, VBM and pupillometry point towards handedness-related differences in the neural mechanisms underlying hemispheric lateralization during face processing in humans.

Throughout history, a strong prevalence of right-handedness has been observed in humans^10^,^29^. Handedness has been suggested to arise from a complex interplay of genetic, hormonal, developmental and cultural factors^30^. Focusing on the role of genetic influences, models were proposed which initially described handedness as a monogenetic trait^15^, whereas more recent approaches stress the relevance of complex interactions among various genes^31^. Appealingly, these genetic models not only provide a description of handedness, but similarly offer predictions about the hemispheric lateralization of language, suggesting a joint molecular basis for handedness and brain asymmetry^16^. In this vein, a link between handedness and hemispheric lateralization of cognitive functions is now well established for language^9^,^10^,^11^, spatial attention^12^, motor processes^13^,^14^, and face processing^17^,^18^.

Here, we replicated these handedness-related differences in the hemispheric lateralization of face-sensitive BOLD activation. Specifically, right- and left-handers differed with regard to the lateralization of the FFA, whereas the OFA was similarly lateralized to the right hemisphere in both groups. This distinction was thought to originate from the spatial proximity of the left FFA with the fusiform word form area and, in particular, from a decreased competition of the representations of faces and words in the left hemisphere in left-handers^18^. Notably, we here extend these descriptive observations by providing a mechanistic explanation of how handedness relates to hemispheric lateralization of face-sensitive activation. Our analyses revealed significant differences between right- and left-handers for a couple of modulatory parameters (but not for endogenous and driving input parameters):

First, we observed handedness-related differences of the face-specific intra- and interhemispheric integration in the core face perception network. In general, behavioral^32^, functional^33^ and effective connectivity studies^24^,^26^ have jointly demonstrated the relevance of both intra- and interhemispheric integration during face processing. Here, we observed enhanced face-specific modulatory influences on the intra- and interhemispheric connections towards the left FFA in left-handers. This suggests that handedness-related differences in the hemispheric lateralization of the FFA are due to a stronger recruitment and thus more decisive role (i.e., up-regulation) of the left FFA in left-handers, rather than a down-regulation of the right FFA during face perception. Such a decisive role of the left FFA during face processing is consistent with those rare studies that tested left-handed prosopagnostic patients and found lesions to be located primarily in the middle fusiform gyrus of the left hemisphere instead of the right^34^,^35^. Critically, the exact functional role of the left FFA in left-handers remains elusive so far. Recent imaging studies have demonstrated a non-negligible role of face-sensitive regions in the left hemisphere and found a fundamental dissociation between right and left FFA – that is, the right FFA is involved in face/non-face judgements whereas the left FFA processes “low-level” face resemblance^36^. Handedness-related differences in the intra- and interhemispheric recruitment of the left FFA might therefore point towards different processing strategies of the two handedness groups during face perception, with left-handers relying more strongly on a low-level (feature-based) strategy. However, as only right-handed subjects were tested in the study by Meng and colleagues^36^, it remains subject to future research whether the above-mentioned dissociation between right and left FFA also applies to left-handers. Combing the generative modelling approach presented here with the paradigm introduced in Meng *et al.*^36^ might offer a promising experimental framework for addressing these questions.

Second, our analyses revealed larger modulatory influences by the right visual field (regardless of the stimulus category) on the intrahemispheric connection from left V1 to left FFA and the interhemispheric connection from left to right FFA in left-handers. This was somewhat unexpected given that we did not observe handedness-related differences in the hemispheric lateralization of BOLD activity for the visual field baseline contrasts. Considering that FFA responds not only to faces but is also activated (to a lesser degree) by other stimuli^37^, the present results suggest that left-handers recruit FFA to a larger amount also for stimulus categories other than faces. This again points to the possibility of different processing strategies in the two handedness groups. However, given the inconsistency between fMRI and DCM results, a more thorough investigation of the handedness-related differences in the modulatory influences of the RVF are essential for a deeper understanding of the mechanisms underlying these differences. This is however beyond the scope of this paper, which focuses on handedness-related differences during face processing.

Third, consistent with the observations at the level of BOLD activity, differences between the two handedness groups are restricted to modulatory influences on the connections from or towards FFA, but not OFA^18^. This suggests that handedness-related differences in hemispheric lateralization result from processes at relatively late stages in face processing. Overall, the present study nicely illustrates the potential of DCM for delineating the putative processes that generated the observed activation patterns^25^ and their lateralization^38^.

As a structural correlate of the more pronounced role of left FFA during face processing in left-handers, we found the gray matter volume in their left fusiform gyrus to be significantly larger as compared to right-handers. This difference might be attributable to local plasticity changes in the left fusiform gyrus, resulting from the enhanced recruitment of the left FFA during face processing in left-handers. Local plasticity of the human brain has been described extensively using VBM^39^. The alternative explanation suggests that differences in the gray matter volume of the left fusiform gyrus might be due to innate predisposition, which would then give rise to the observed differences at the level of BOLD activity and effective connectivity. However, given the present data, assertions regarding the exact nature of the causes of the observed anatomical differences remain speculative. In addition to the left fusiform gyrus, we found clusters of increased gray matter volume in the left middle cingulate gyrus and the right precuneus, which have been related to language processing^40^ and to aspects of visuospatial processing^41^, in particular in the coordination of motor behavior^42^. At a more liberal statistical threshold, larger gray matter volume in left- as compared to right-handers was also observed bilaterally in the supplementary motor area, a region involved in regulating all sorts of motor actions and crucial for initiating hand movements^43^. The latter is consistent with previously reported effects of handedness on brain anatomy, which have mainly been described in terms of cerebral asymmetries, and have been demonstrated for the motor cortex^44^, the primary somatosensory cortex^45^, and the planum temporale^46^. On the contrary, Good *et al.* found no evidence for structural correlates of handedness^47^. These somewhat inconsistent findings in the literature (and the present study) might in part be attributable to the sample characteristics of the respective studies. Especially for studies with small sample sizes (as is the case in the present work), individual anatomical properties in this sample have a large impact on the overall effect^48^. Given that, for the present sample, fMRI and DCM analyses suggested a more decisive role of the left FFA in left-handers, handedness-related differences in gray matter volume in the left fusiform gyrus might also be more prominent and thus become significant. To assess whether the observed differences in brain anatomy truly generalize to the whole population, we aim to replicate in a future study our findings in a large sample of right- and left-handers. Such a large-scale study will hopefully contribute to a better understanding of the relationship between handedness, brain function and brain anatomy.

As a physiological measure of handedness-related differences in the mechanisms underlying face processing, an exploratory analysis suggested that pupil dynamics (representing slow changes in pupil size over the blocks; see Methods for detailed information) varied between right- and left-handers when processing faces, but not when processing objects or scrambled images. While pupil dynamics are primarily associated with changes in low-level image features^49^, the pupil also provides some capacity for mapping cognitive states and cognitive processing. As such, measures of pupil dynamics have recently been combined with fMRI to provide exciting new insights into brain function^50^. Handedness-related differences in pupil dynamics might therefore be associated with differences in the cognitive processes related to face processing. The following two interpretations (or a combination of both) seem most plausible and integrate well with the DCM and VBM results: First, a more feature-based (as opposed to holistic) processing strategy in left-handers would explain the diminished pupil constrictions^24^,^51^ and at the same time account for the enhanced recruitment of the left FFA (where low-level face analysis is performed^36^) and its increased gray matter volume. Second, enhanced cognitive effort during face processing in left-handers would explain the diminished pupil constrictions^52^, as well as the overall enhanced modulatory connectivity in the core face perception network and the larger gray matter volume in face-sensitive cortex. Such enhanced cognitive effort for left-handers has long been suggested and controversially discussed in the community^53^. We would like to emphasize that from the present exploratory analyses the exact nature of handedness-related differences in pupil dynamics remain ambiguous and our interpretations therefore speculative. Future analyses will need to delineate more carefully which process most likely underlies the observed effects. Nevertheless – and most importantly – our findings support the notion that handedness relates to differences in the organization of the core face perception network, consistent with the effects found at the level of BOLD activity, effective connectivity, and brain anatomy.

In a broader context, our findings might provide evidence for the entanglement of brain networks that are structurally and functionally remote. Specifically, lateralization of one brain function, such as handedness (representing lateralization of motor processes), relates to hemispheric lateralization of other distinct cognitive functions^8^, such as language^9^,^11^, spatial attention^12^, and face perception^17^,^18^. This interaction of distant brain networks indicates that an as yet concealed broader process – potentially mediated by a complex interplay of various factors, including genetic influences^8^,^30^ – regulates the functional and anatomical organization of hemispheric lateralization in the human brain. The framework introduced in the present study offers a promising avenue for systematically investigating how handedness relates to the hemispheric lateralization of the human brain and how lateralization of individual cognitive functions might influence each other^54^. Specifically, applying this multimodal approach in a more comprehensive study which probes different lateralized cognitive functions at the same time (e.g., language, spatial attention, face processing) in a large sample of healthy subjects will provide a powerful approach to shed light on the underlying processes.

Notably, the present study is also subject to some limitations: First, we only considered the core regions (OFA, FFA) involved in face processing, thus neglecting additional regions such as pSTS, amygdala or insula^1^. This was motivated by work indicating that these additional regions serve face-related functions, but are not necessary for pure face detection^55^. Second, we defined one FFA per hemisphere, although multiple face-sensitive regions in the fusiform gyrus have recently been suggested^56^. Critically, as the exact role of these regions is largely unknown, no well-founded hypotheses on their interplay exist so far. Nevertheless, the present framework provides an ideal starting point to establish more sophisticated experimental paradigms which will enable the development of bilateral models covering a more extended network, embedding additional face-sensitive regions (e.g., pSTS) and addressing the questionable simplicity of FFA.

In conclusion, our results provide novel insights into the association of handedness with differences in the organization of the core face perception network based on a multimodal approach combining conventional activation measures with effective connectivity, brain anatomy and pupillometry. In this framework, our results indicate diverse neural mechanisms underlying face processing in right- as opposed to left-handers and specifically point towards a more pronounced recruitment of the left FFA in left-handers. From a more general perspective, these data provide evidence for the entanglement of structurally and functionally remote brain networks, tentatively suggesting a broader underlying process regulating hemispheric lateralization.

## Materials and Methods

### Subjects

Forty healthy subjects with no history of neurological or psychiatric diseases participated in this study. Half of them were right-handed (12 females, age range: 19–29 years, mean: 23.2 ± 2.5 years), and half were left-handed (12 females, age range: 20–30 years, mean: 24.3 ± 2.7 years), according to the Edinburgh Handedness Inventory (EHI) with a cut-off at ±30. The right-handed subjects were taken from a larger sample which was included in a previous study^26^. Subjects were selected such that groups matched with regard to the ratio of male and female subjects (i.e., 8/12), age (*p* = 0.17), and absolute EHI score (*p* = 0.11). All subjects had normal or corrected-to-normal vision. They gave written informed consent prior to the experiment, which was performed in accordance with the Declaration of Helsinki and approved by the local ethics committee of the Medical Faculty of the University of Marburg.

### Experimental procedure

Subjects viewed either gray-scale neutral faces (“F”), objects (“O”) or scrambled images (“S”) in the left (“LVF”) or right visual field (“RVF”) in a blocked design while fixating a cross in the center of a screen^26^. Scrambled images were the Fourier-randomized versions (i.e., randomizing the phase component of the Fourier transform) of the face and objects stimuli. In particular, half of the scrambled images were generated as the Fourier-randomized versions of the face stimuli, whereas the other half of the scrambled images were generated as the Fourier-randomized versions of the object stimuli. All stimuli were presented as circular patches (radius: 2.17 degree) on an MRI-compatible LCD screen (LG SL9000, 60 Hz, 4:3, 1024 × 786 pix) using Presentation 11.0 (Neurobehavioral Systems, Albany, CA, USA, http://www.neurobs.com/). The center of the circular patches was located 4.02 degree lateral to the fixation cross. Proper fixation was controlled for by recording the direction of eye gaze using an MRI-compatible infrared-sensitive camera (EyeLink 1000, SR Research, Osgoode, ON, Canada).

To test for any confounds due to subjects’ fixation, differences in eye gaze between right- and left-handers and between the different experimental conditions were assessed. To this end, the mean gaze eccentricity was calculated separately for each experimental condition and subject. Individual eccentricity values were then entered into a 3-way repeated measures ANOVA (between-subject factor: handedness, within-subject factor: stimulus, hemifield) in IBM SPSS Statistics 20 (Armonk, NY: IBM Corp. Released 2011). The ANOVA revealed no significant main effect of handedness (*F*_(1,38)_ < 0.01, *p* = 0.98), stimulus (*F*_(2,37)_ = 0.84, *p* = 0.44), or hemifield (*F*_(2,38)_ = 1.86, *p* = 0.18). Similarly, there was no significant effect for the handedness × stimulus (*F*_(2,37)_ = 2.09, *p* = 0.14), handedness × hemifield (*F*_(2,38)_ = 2.71, *p* = 0.11), stimulus × hemifield (*F*_(2,37)_ = 2.41, *p* = 0.10), or handedness × stimulus × hemifield interaction (*F*_(2,37)_ = 0.45, *p* = 0.64). Thus, the effects of handedness discussed in this paper cannot be attributed to differences in subjects’ fixation patterns.

Stimuli were presented in blocks with a duration of 14.5 s (i.e., 10 functional whole-brain scans), interleaved with blank periods of the same length in which only the fixation cross was shown. Each stimulus was shown for 150 ms with an inter-stimulus interval (ISI) of 250 ms. Within each block, stimuli from one of the six conditions (i.e., faces, objects or scrambled images, each either in the left or right visual field) were shown. Each condition was performed 7 times in a pseudo-randomized order; hence, the experiment comprised 84 blocks (i.e., 42 stimulus blocks, 42 fixation blocks). To avoid fatigue, the experiment was divided into three parts, with successive parts being interleaved by 60.9 s (i.e., 42 functional whole-brain scans) of rest. Subjects remained inside the scanner during these breaks and were not allowed to move as the scanner continued to run (total experiment length: ~23 min).

### Image acquisition

Imaging data were acquired with a 12-channel head matrix receive coil on a 3-Tesla MR scanner (Siemens TIM Trio, Erlangen, Germany) at the Department of Psychiatry, University of Marburg. A T_2_^*^-weighted single-shot gradient-echo echo-planar-imaging sequence (EPI) provided 940 functional images sensitive to the Blood Oxygen Level Dependent (BOLD) contrast (30 slices, TR = 1450 ms, TE = 25 ms, matrix size 64 × 64 voxels, voxel size 3 × 3 × 4 mm^3^, FoV = 192 × 192 mm^2^, flip angle 90°). Slices were acquired in descending order parallel to the intercommissural (AC-PC) plane. Additionally, a T1-weighted volume covering the whole brain was obtained using a magnetization-prepared rapid gradient-echo (3d MP-RAGE) sequence in sagittal plane (176 slices, TR = 1900 ms, TE = 2.26 ms, matrix size 256 × 256 voxels, voxel size 1 × 1 × 1 mm^3^, FoV = 256 × 256 mm^2^, flip angle 9°).

### Image data processing

Functional images were analyzed using SPM8 (v4290; Statistical Parametric Mapping, Wellcome Trust Centre for Neuroimaging, London, UK; http://www.fil.ion.ucl.ac.uk) and Matlab (Mathworks, Natick, MA, USA). The first four scans were discarded from the analysis. The remaining functional images were realigned to the mean image, coregistered with the high-resolution anatomical image, normalized to the MNI standard space using the spatial normalization parameters obtained from the unified segmentation-normalization approach to the anatomical image, resampled to a voxel size of 2 × 2 × 2 mm^3^, and smoothed with an isotropic 6-mm full-width at half-maximum (FWHM) Gaussian kernel. For each subject, statistical analysis of the preprocessed images was then performed by means of a first-level General Linear Model. Each condition (i.e., “F_LVF”, “F_RVF”, “O_LVF”, “O_RVF”, “S_LVF”, and “S_RVF”) was modeled as a block regressor, convolved with SPM’s hemodynamic response function. Furthermore, the six realignment parameters were introduced as nuisance regressors to control for movement-related artifacts. Finally, low-frequency noise in the data was accounted for by a high-pass filter (cut-off frequency: 1/128 Hz).

For each subject, voxels were identified that responded more strongly to faces than to objects and scrambled images, regardless of the visual field (“[2*F]-[O + S]”). Additionally, activation to stimuli presented in the left visual field and in the right visual field was identified from the baseline contrasts “LVF” and “RVF”, respectively. The individual contrast images were then entered into random effects group analyses (one-sample *t*-tests). Group-level activations were thresholded at *p* < 0.001, uncorrected and anatomically localized using the Anatomy toolbox extension within SPM.

The hemispheric lateralization of face-sensitive BOLD activation was investigated by means of the lateralization index (LI). To this end, regions of interest (ROI) were defined for OFA and FFA, each in both hemispheres, from the individual face-sensitive contrast images ([2*F]-[O + S]). In line with previous approaches^24^,^26^, individual center coordinates of the ROIs were manually defined as the subject-specific maxima close to the respective group-level peak activation under the following anatomical constraints: OFA had to be located in the inferior occipital gyrus and FFA in the fusiform gyrus. ROIs were then defined as a 4-mm sphere centered on the individual coordinates. Using the Bootstrap procedure implemented in the LI toolbox extension within SPM, the degree of hemispheric lateralization was assessed separately for OFA and FFA.

Additionally, to test whether handedness-related differences in hemispheric lateralization of BOLD activation were restricted to face perception rather than to overall object perception, ROIs were defined for left and right lateral occipital complex (LOC)^57^ from the individual object-sensitive contrast images ([O]-[S]). Lateralization of object perception was then assessed using the Bootstrap procedure within the LI toolbox.

### Dynamic causal modeling

Dynamic causal modeling (DCM)^25^ is a frequently used Bayesian framework for investigating the effective connectivity (i.e., directed interactions) in neural networks and how these interactions are perturbed by experimental manipulations. In this regard, DCM describes the dynamics of the neuronal states by means of a bilinear differential equation:


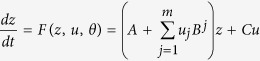


where z represents the neuronal states, A defines the endogenous connection strengths, B^j^ describes the influences of the experimental manipulation u_j_ on the endogenous connections (modulatory connectivity), and C quantifies how experimental manipulations directly influence the neuronal states (driving inputs). Forwarding the predicted neuronal signal through a hemodynamic model yields predictions of the BOLD signal time series within each network region. Using a Variational Bayes approach under Gaussian assumptions on the prior and posterior distributions (Laplace approximation), the sufficient statistics of the posterior densities of the model parameters (i.e., conditional mean and covariance) can be estimated by maximizing the negative free energy. At the same time, this approach tightens the negative free energy as a lower bound approximation to the log model evidence.

#### Time series extraction

The subject-specific face-sensitive ROIs (OFA and FFA, each in both hemispheres), which were defined for assessing hemispheric lateralization of face-sensitive activation (see above), were entered into the DCM analyses. Additionally, two ROIs were defined in the left and right early visual cortex (EVC), most likely representing primary visual cortex V1, from the visual field baseline contrast (RVF/LVF), respectively. Again, individual ROI center coordinates were defined as the subject-specific maximum within left and right Brodmann Area 17 close to the group-level maxima. For each subject and each of the six ROIs, BOLD signal time series were then extracted as the first eigenvariate of all activated voxels (*p* < 0.05, uncorrected). An effects-of-interest *F*-contrast was used to mean-center the time series and remove movement-related variance.

#### Definition of the model space

Since the exact mechanisms underlying the neuronal dynamics of face processing are *a priori* unknown, a set of different models was defined, representing distinct hypotheses on the effective connectivity among the ROIs considered in this study. For all models, the endogenous connectivity and driving inputs (i.e., A- and C-matrix) were identical. All three regions within each hemisphere (i.e., EVC, OFA and FFA) were connected via reciprocal endogenous intrahemispheric connections. The hierarchical organization of the core face perception network from early visual areas to FFA via OFA is well established^1^. Moreover, recent studies have emphasized the possibility of direct connections from early visual areas to the FFA, bypassing the OFA^58^. This route has been suggested to mediate lower-level categorization abilities such as discriminating faces from objects. Additionally, reciprocal interhemispheric connections were set between the homotopic face-sensitive regions of both hemispheres^59^,^60^. Notably, interhemispheric connections between heterotopic regions and between bilateral EVC are less pronounced in humans^60^, and were thus not included in the present models. A more comprehensive motivation for this choice of model structure can be found in our previous work^24^.

To specify the modulatory influences and driving inputs of the DCMs, a new GLM (different from the one used for the conventional fMRI analyses) was created that included the regressors for the DCM analysis. This GLM contained the following 5 block regressors: “RVF” (modelling all stimuli presented in the right visual field, regardless of the stimulus category), “LVF” (modelling all stimuli presented in the left visual field, regardless of the stimulus category), “faces” (modelling all face stimuli, regardless of the visual field), “faces|RVF” (modelling all face stimuli presented in the right visual field), and “faces|LVF” (modelling all face stimuli presented in the left visual field).

Since bilateral EVC served as the input regions of the DCMs, driving inputs thus modulated neuronal activity in the contralateral EVC (i.e., LVF modulated right EVC, RVF modulated left EVC).

Experimental manipulations were then allowed to modulate the intra- and interhemispheric endogenous connections. In line with previous approaches^20^,^21^,^24^, modulatory influences on the intrahemispheric connections were restricted to the forward connections. Notably, models differed with regard to their exact modulatory connectivity patterns. The differences between the competing models could be expressed by three factors: 1) the site of modulatory effects on intrahemispheric connections, 2) the context of modulatory inputs on intrahemispheric connections, and 3) the context of modulatory inputs on interhemispheric connections (Fig. 1).

We created six different model families^27^, grouping models with the same site of experimental perturbations on intrahemispheric forward connections. Families varied from DCMs with modulations on only one forward connection per hemisphere (e.g., family A) to a family where all six forward connections were modulated (family F). Within each family, models differed with regard to the context of the modulatory influences on the intra- and interhemispheric connections. Each connection could be modulated either by 1) the visual field of the stimulus (S), 2) the perception of faces (F), 3) both the perception of faces and the visual field (F + S), or 4) the perception of faces conditional on the visual field (F × S). These alternatives exist equally for intra- and interhemispheric connections. This approach followed previous work on intra- and interhemispheric integration during lateralized cognitive processes^24^,^61^. For each subject, this resulted in 16 models within each family and thus 96 models in total. An exemplary model (model S/F, family F) is shown in Fig. 1 (bottom, right). Furthermore, we provide a more detailed illustration of all 16 models of family F in the Supplementary Information (Supplementary Fig. S4).

Experimental inputs were specified as block regressors, which were not mean-centered – that is, the endogenous parameter estimates represent the connection strengths in the absence of experimental manipulations. Model inversion was then performed using DCM8 as implemented in SPM8 (v4290). There were two reasons for relying on this particular software version: First, for more recent DCM versions (i.e., less-regularizing priors on the model parameters), exploratory analyses had indicated that models would no longer converge under the default upper bound on iterations (i.e., 128 iterations) when considering connections (and their modulations) from EVC to both OFA and FFA^24^. Second, a recent study showed high test-retest reliability of both Bayesian model section and model parameter estimation for this DCM version (and the inherent tight shrinkage priors^62^) using the face perception paradigm of this study^26^.

#### Bayesian model averaging

Individual parameter estimates were calculated using random effects Bayesian model averaging (BMA)^27^ across all 96 models – that is, all models of all families were potentially considered for BMA. Notably, however, to guarantee computational efficiency of the estimation, only those models that fell in the standard Occam’s window with a posterior odds ratio of *p* > 0.05 were actually included in the BMA procedure. This means, only those models *m*_*i*_ contributed to the averaged (BMA) parameter estimates that satisfied the following criterion


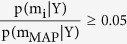


The individual BMA parameter estimates were then entered into group-level inferences. First, effective connectivity patterns were assessed separately for right- and left-handers. The significance of each parameter was tested by a one-sample *t*-test at a statistical threshold of *p* < 0.05 (false discovery rate (FDR)-corrected for multiple comparisons). Second, differences in the parameter estimates between right- and left-handers were estimated by means of two-sample *t*-tests (*p* < 0.05, FDR-corrected, within each parameter class).

### Voxel-Based Morphometry

For each subject, gray matter (GM) volume maps were extracted from the high-resolution anatomical image using the VBM8 toolbox (http://dbm.neuro.uni-jena.de/vbm). Anatomical images were processed according to the standard DARTEL approach, which includes tissue segmentation, bias correction, normalization to the MNI standard space and spatial smoothing with an isotropic 6-mm FWHM Gaussian kernel. To allow for inferences on the relative GM volume, the GM volume maps were corrected for non-linear warping only. Significant differences in the relative GM volume between right- and left-handers were assessed by means of a between-subject ANOVA at a cluster extent threshold of *p* < 0.05, family-wise error (FWE)-corrected (voxel level threshold of *p* < 0.001, uncorrected). Covariates for “age” and “gender” were introduced to control for variance in gray matter volume related to these variables.

### Pupillometry

Pupil size (i.e., pupil diameter) was recorded at a rate of 500 Hz using the EyeLink system. Individual pupil size traces were preprocessed as follows: First, blinks were detected by the EyeLink software and then interpolated using cubic spline interpolation. Second, the interpolated traces were z-normalized over the whole session to enable comparison across subjects. Third, high-frequency noise in the pupil size traces was suppressed using a sliding square window of 50 ms width. To characterize the change in pupil size (i.e., pupil dynamics) for each block by a single value, the average pupil size during the first 50 ms of each block (“baseline”) was subtracted from the average value during a late time interval from 9 s to 11 s after block onset. For each subject, condition-averaged values for this slow change in pupil size were calculated and then entered into a 3-way repeated measures ANOVA (between-subject factor: handedness, within-subject factor: stimulus, hemifield) in IBM SPSS Statistics 20. Post-hoc *t*-tests were then implemented to delineate more closely the observed main effect of stimulus, as well as the handedness × stimulus interaction.

## Additional Information

**How to cite this article**: Frässle, S. *et al.* Handedness is related to neural mechanisms underlying hemispheric lateralization of face processing. *Sci. Rep.* **6**, 27153; doi: 10.1038/srep27153 (2016).

## Supplementary Material

Supplementary Data

## Figures and Tables

**Table 1:** Coordinates of brain regions displaying significant face-sensitive activations ([2*F]-[O + S]) for right- and left-handers.

Cortical region	Hemisphere	MNI coordinates			Cluster size (voxels)	Z-score
		x	y	z		
*Right-handers*						
Fusiform gyrus	R	42	−58	−20	737	5.47
Fusiform gyrus	R	40	−44	−18		5.37
Middle temporal	R	48	−64	12		4.22
Inferior occipital	R	48	−76	−2		4.08
Inferior occipital	R	36	−74	−18		4.06
Precentral gyrus	R	44	−6	50	228	4.67
Amygdala	L	−26	−10	−14	89	4.29
Middle frontal	R	36	42	22	62	4.19
Cerebellum	R	8	−58	−8	88	4.17
Suppl. motor area	R	6	2	52	41	3.92
Fusiform gyrus	L	−40	−50	−28	23	3.86
Amygdala	R	−22	−6	−16	41	3.80
Suppl. motor area	L	−6	−4	64	29	3.57
Fusiform gyrus	L	−40	−64	−18	25	3.45
Superior temporal	R	54	−36	12	22	3.40
Inferior occipital	L	−36	−82	−12	7	3.27
*Left-handers*						
Superior temporal	R	50	−40	12	272	5.24
Middle temporal	R	68	−44	8		4.33
Middle temporal	R	56	−62	14	194	4.74
Inferior occipital	R	42	−80	−10	161	4.62
Fusiform gyrus	R	40	−44	−20	233	4.60
Fusiform gyrus	L	−42	−50	−24	118	4.04
Fusiform gyrus	R	38	−60	−14		3.95
Fusiform gyrus	L	−38	−62	−18		3.93
Middle temporal	L	−42	−68	18	38	3.93
Inferior temporal	L	−44	−20	−20	22	3.83
Amygdala	R	20	−2	−16	19	3.80
Amygdala	L	−22	−10	−14	10	3.58

Shown are brain regions where activation is greater during the perception of faces as compared to the perception of objects and scrambled images. For all regions, MNI coordinates, cluster sizes and Z-scores are reported. Results are thresholded at a voxel-level threshold of *p* < 0.001, uncorrected.

**Table 2:** Endogenous connectivity parameter estimates for right- and left-handers.

	right-handers (RH)		left-handers (LH)		difference (RH vs. LH)	
	mean ± std	*p*	mean ± std	*p*	*p*	*p_corr*
FFA_L → FFA_R	**0.228** **±** **0.147**	**1.29e-6**	**0.253** **±** **0.087**	**6.95e-11**	5.10e-1	7.43e-1
FFA_L → OFA_L	**0.106** **±** **0.096**	**8.78e-5**	**0.147** **±** **0.095**	**1.36e-6**	1.90e-1	5.08e-1
FFA_L → EVC_L	**−0.111** **±** **0.222**	**3.83e-2**	**−0.141** **±** **0.206**	**6.30e-3**	6.52e-1	7.50e-1
FFA_R → FFA_L	**0.216** **±** **0.133**	**6.98e-7**	**0.235** **±** **0.123**	**6.40e-8**	6.35e-1	7.50e-1
FFA_R → OFA_R	**0.124** **±** **0.147**	**1.30e-3**	**0.197** **±** **0.141**	**5.40e-6**	1.17e-1	3.40e-1
FFA_R → EVC_R	−0.005 ± 0.158	9.00e-1	−0.061 ± 0.213	2.15e-1	3.45e-1	6.90e-1
OFA_L → OFA_R	**0.214** **±** **0.125**	**3.08e-7**	**0.185** **±** **0.154**	**3.47e-5**	5.11e-1	7.43e-1
OFA_L → FFA_L	**0.155** **±** **0.127**	**2.92e-5**	**0.051** **±** **0.092**	**2.25e-2**	5.10e-3	8.15e-2
OFA_L → EVC_L	**−0.233** **±** **0.140**	**4.78e-7**	−0.126 ± 0.272	5.23e-2	1.25e-1	3.40e-1
OFA_R → OFA_L	**0.209** **±** **0.093**	**4.64e-9**	**0.217** **±** **0.165**	**1.15e-5**	8.60e-1	8.60e-1
OFA_R → FFA_R	**0.149** **±** **0.117**	**1.66e-5**	**0.125** **±** **0.084**	**2.19e-6**	4.53e-1	7.43e-1
OFA_R → EVC_R	**−0.177** **±** **0.222**	**2.10e-3**	−0.099 ± 0.225	6.29e-2	2.82e-1	6.43e-1
EVC_L → FFA_L	**0.110** **±** **0.078**	**4.77e-6**	**0.155** **±** **0.069**	**4.55e-9**	6.24e-1	3.33e-1
EVC_L → OFA_L	**0.220** **±** **0.119**	**9,71e-8**	**0.213** **± 0.106**	**2.88e-8**	8.43e-1	8.60e-1
EVC_R → FFA_R	**0.121** **±** **0.073**	**4.87e-7**	**0.180** **±** **0.105**	**3.12e-7**	4.34e-1	3.33e-1
EVC_R → OFA_R	**0.194** **±** **0.069**	**1.23e-10**	**0.182** **±** **0.090**	**2.40e-8**	6.57e-1	7.50e-1

Endogenous parameter estimates are provided in terms of their mean and standard deviation (i.e., mean ± std), and were estimated using BMA over all models within the standard Occam’s window (*p* < 0.05). Significant connections at the group level (*p* < 0.05, FDR-corrected) are printed bold (*columns 2–5*). Additionally, for each parameter, uncorrected and corrected p-values of the two-sample *t*-tests on handedness-related differences in the endogenous parameter estimates are provided. Connections showing a significant handedness-related difference (*p* < 0.05, FDR-corrected) are printed bold (*columns 6–7*). RH = right-handers, LH = left-handers.

**Table 3:** Modulatory parameter estimates for right- and left-handers.

	right-handers (RH)		left-handers (LH)		difference (RH vs. LH)	
	mean ± std	*p*	mean ± std	*p*	*p*	*p_corr*
*modulatory parameters (RVF)*						
FFA_L → FFA_R	**0.061** **±** **0.104**	**1.67e-2**	**0.180** **±** **0.065**	**1.47e-10**	**1.00e-4**	**3.30e-3**
OFA_L → OFA_R	**0.110** **±** **0.176**	**1.19e-2**	0.016 ± 0.114	5.33e-1	5.34e-2	3.21e-1
OFA_L → FFA_L	**−0.063** **±** **0.106**	**1.59e-2**	−0.068 ± 0.144	4.74e-2	8.93e-1	8.92e-1
EVC_L → FFA_L	0.018 ± 0.055	1.67e-1	**0.091** **±** **0.089**	**1.99e-4**	**3.10e-3**	**3.08e-2**
EVC_L → OFA_L	**0.119** **±** **0.095**	**2-06e-5**	**0.106** **±** **0.119**	**7.35e-4**	7.13e-1	8.74e-1
*modulatory parameters (LVF)*						
FFA_R → FFA_L	**0.100** **±** **0.105**	**4.44e-4**	**0.149** **±** **0.101**	**2.42e-6**	1.38e-1	4.06e-1
OFA_R → OFA_L	**0.108** **±** **0.118**	**7.19e-4**	**0.077** **±** **0.133**	**1.84e-2**	4.64e-1	6.64e-1
OFA_R → FFA_R	**−0.116** **±** **0.105**	**8.52e-5**	**−0.068** **±** **0.093**	**4.00e-3**	1.33e-1	4.06e-1
EVC_R → FFA_R	−0.005 ± 0.044	6.18e-1	0.013 ± 0.101	5.66e-1	4.65e-1	6.64e-1
EVC_R → OFA_R	**0.113** **±** **0.100**	**7.06e-5**	0.057 ± 0.144	9.16e-2	1.60e-1	4.06e-1
*modulatory parameters (faces)*						
FFA_L → FFA_R	**0.169** **±** **0.105**	**7.38e-7**	**0.176** **±** **0.070**	**6.46e-10**	8.13e-1	8.74e-1
FFA_R → FFA_L	**0.060** **±** **0.063**	**4.67e-4**	**0.128** **±** **0.081**	**1.08e-6**	**5.35e-3**	**4.03e-2**
OFA_L → OFA_R	**0.154** **±** **0.100**	**1.43e-6**	**0.149** **±** **0.113**	**1.16e-5**	8.66e-1	8.93e-1
OFA_L → FFA_L	**0.094** **±** **0.071**	**1.07e-5**	**0.064** **±** **0.075**	**1.10e-3**	2.10e-1	4.51e-1
OFA_R → OFA_L	**0.087** **±** **0.050**	**2.65e-7**	**0.075** **±** **0.072**	**1.64e-4**	5.37e-1	7.01e-1
OFA_R → FFA_R	**0.120** **±** **0.077**	**1.18e-6**	**0.076** **±** **0.085**	**7.15e-4**	9.89e-2	4.06e-1
EVC_L → FFA_L	**0.070** **±** **0.090**	**2.40e-3**	**0.165** **±** **0.083**	**3.46e-8**	**1.40e-3**	**2.03e-2**
EVC_L → OFA_L	**0.137** **±** **0.105**	**1.27e-5**	**0.128** **±** **0.087**	**2.83e-6**	7.55e-1	8.74e-1
EVC_R → FFA_R	**0.065** **±** **0.092**	**5.60e-3**	**0.105** **±** **0.077**	**6.88e-6**	1.40e-1	4.06e-1
EVC_R → OFA_R	**0.129** **±** **0.068**	**6.51e-8**	**0.136** **±** **0.111**	**2.83e-5**	8.13e-1	8.74e-1
*modulatory parameters (faces|RVF)*						
FFA_L → FFA_R	0.003 ± 0.008	1.71e-1	–	–	1.62e-1	4.06e-1
OFA_L → OFA_R	0.001 ± 0.003	1.65e-1	–	–	1.57e-1	4.06e-1
OFA_L → FFA_L	−0.000 ± 0.001	9.01e-2	−0.000 ± 0.001	3.30e-1	1.94e-1	4.48e-1
EVC_L → FFA_L	0.002 ± 0.005	6.47e-2	0.001 ± 0.003	1.65e-1	3.66e-1	5.78e-1
EVC_L → OFA_L	–	–	0.000 ± 0.001	3.63e-1	3.57e-1	5.78e-1
*modulatory parameters (faces|LVF)*						
FFA_R → FFA_L	0.001 ± 0.005	2.38e-1	–	–	2.31e-1	4.61e-1
OFA_R → OFA_L	0.002 ± 0.008	3.31e-1	–	–	3.25e-1	5.74e-1
OFA_R → FFA_R	0.000 ± 0.001	3.48e-1	0.000 ± 0.002	3.30e-1	8.16e-1	8.74e-1
EVC_R → FFA_R	0.002 ± 0.005	9.30e-2	0.001 ± 0.004	1.65e-1	4.99e-1	6.81e-1
EVC_R → OFA_R	–	–	0.001 ± 0.002	3.23e-1	3.16e-1	5.74e-1

Modulatory parameter estimates are provided in terms of their mean and standard deviation (i.e., mean ± std), and were estimated using BMA over all models within the standard Occam’s window (*p* < 0.05). Significant modulatory influences at the group level (*p* < 0.05, FDR-corrected) are printed bold (*columns 2–5*). Additionally, for each parameter, uncorrected and corrected p-values of the two-sample *t*-tests on handedness-related differences in the modulatory parameter estimates are provided. Modulatory influences showing a significant handedness-related difference (*p* < 0.05, FDR-corrected) are printed bold (*columns 6–7*). RH = right-handers, LH = left-handers.
