# Supplementary material for: Handedness is related to neural mechanisms underlying hemispheric lateralization of face processing
Source: Sci Rep. 2016 Jun 2;6:27153. doi: 10.1038/srep27153 (PMC4890016; doi:10.1038/srep27153)
Supplement: Supplementary Data [file srep27153-s1.doc]

**Supplementary Information**

**Handedness is related to neural mechanisms underlying
hemispheric lateralization of face processing**Stefan Frässle, Sören Krach, Frieder Michel Paulus & Andreas Jansen

**Supplementary Note S1: Specificity of handedness-related differences in hemispheric lateralization**

To delineate the nature of the handedness-related differences in hemispheric lateralization more closely, we performed additional analyses targeted to the following questions: First, are handedness-related differences in the hemispheric lateralization of OFA and FFA restricted to face processing or can similar effects be observed for the visual field baseline contrasts (i.e., LVF, RVF) as well? Second, are handedness-related differences restricted to the face-sensitive representations (i.e., core face perception network) or do these effects represent an overall lateralization change in object perception (i.e., in object-sensitive cortex)?

To address the first question, we computed for each subject an LI for OFA and FFA for the LVF and RVF baseline contrast, separately (Supplementary Fig. S1a+b). For neither of the two contrasts, we found a significant handedness×region interaction (LVF: *F*_(1,38)_ = 0.10, *p* = 0.75; RVF: *F*_(1,38)_ = 0.35, *p* = 0.56). Post-hoc two-sample *t*-tests corroborated that there were no handedness-related differences in the hemispheric lateralization of BOLD activation to the visual fields for the OFA (LVF: *t*_(38)_ = 0.06, *p* = 0.95; RVF: *t*_(38)_ = 0.79, *p* = 0.43) or the FFA (LVF: *t*_(38)_ = 0.43, *p* = 0.67; RVF: *t*_(38)_ = -0.16, *p* = 0.87). Additionally, we investigated the univariate responses of the four face-sensitive regions to the visual fields by computing the mean contrast values for the LVF and RVF baseline contrast, respectively (Supplementary Fig. S1c+d). Consistent with the analysis for the LI, the 2×2 mixed effects ANOVAs (between-subject factor: handedness, within-subject factor: hemisphere) revealed no significant handedness×hemisphere interaction for the OFA (LVF: *F*_(1,38)_ = 0.06, *p* = 0.81; RVF: *F*_(1,38)_ < 0.01, *p* = 0.99) or the FFA (LVF: *F*_(1,38)_ = 0.01, *p* = 0.94; RVF: *F*_(1,38)_ = 1.81, *p* = 0.19).

With regard to the second question, we computed for each subject an LI for the lateral occipital complex (LOC; see Methods for detailed information). We found left-hemispheric lateralization of object-sensitive activation in right-handers (mean and std: LI = 0.12 ± 0.24; *t*_(19)_ = 2.24, *p* = 0.04) and left-handers (LH = 0.10 ± 0.22; *t*_(19)_ = 2.03, *p* = 0.06), with no significant difference between the two handedness groups as suggested by a two-sample *t*-test (*t*_(38)_ = 0.31, *p* = 0.76). Again, we checked for handedness-related differences in the univariate responses of left and right LOC. Computing the mean contrast values for the linear contrast [O]-[S] and utilizing a 2×2 mixed effects ANOVA (between-subject factor: handedness, within-subject factor: hemisphere), we found no significant handedness×hemisphere interaction for the LOC (*F*_(1,38)_ = 1.82, *p* = 0.19).

Overall, these results suggest that handedness-related differences in hemispheric lateralization are restricted to face-sensitive activations in the core face perception network – more precisely, in the FFA.

**Supplementary Note S2: Re-analysis under the face-specific conjunction [F>O] ∩ [F>S]**

In the main analysis of the present study, we used the face-sensitive linear contrast [2*F]-[O+S] to identify the regions of interest (ROIs) for our analyses on the relation between handedness and hemispheric lateralization of BOLD activation, as well as between handedness and effective connectivity in the core face perception network. We here repeated these analyses when using the face-selective conjunction [F-O] ∩ [F-S]. As will become apparent below, the results were highly consistent across the two analyses.

*Handedness-related differences in the hemispheric lateralization of face-sensitive activation in OFA and FFA*

In line with our observations from the main analysis (i.e., for the linear contrast [2*F]-[O+S]), the 2×2 mixed effects ANOVA (between-subject factor: handedness, within-subject factor: region) on the LI values of face-sensitive activation revealed a significant handedness×region interaction (*F*_(1,38)_ = 8.06, *p* < 0.01; Supplementary Fig. S2a). Post-hoc two-sample *t*-tests suggested that the FFA was significantly more lateralized to the right hemisphere in right-handers compared to left-handers (*t*_(38)_ = -4.01, *p* < 0.01), whereas the OFA did not show any handedness-related differences (*t*_(38)_ = 0.10, *p* = 0.92). Furthermore, we repeated our analyses on the univariate responses of the four face-sensitive regions. To this end, we estimated the mean contrast values for the conjunction [F-O] ∩ [F-S] in each of the four subject-specific ROIs. Using two separate 2×2 mixed effects ANOVAs (between-subject factor: handedness, within-subject factor: hemisphere), we confirmed our observations from the main analysis that there is no significant handedness×hemisphere interaction for the OFA (*F*_(1,38)_ = 0.01, *p* = 0.91; Supplementary Fig. S2b). Post-hoc two-sample *t*-tests again showed no handedness-related differences in face-sensitive activation for the left OFA (*t*_(38)_ = ‑0.37, *p* = 0.72) or the right OFA (*t*_(38)_ = -0.35, *p* = 0.73). Conversely, a significant handedness×hemisphere interaction was observed for the FFA (*F*_(1,38)_ = 18.59, *p* < 0.01; Supplementary Fig. S2c). Post-hoc two-sample *t*-tests did not reveal handedness-related differences for the left FFA (*t*_(38)_ = -1.56, *p* = 0.13), but for the right FFA (*t*_(38)_ = 2.02, *p* < 0.01).

*Handedness relates to the effective connectivity in the face perception network*

In a next step, we repeated our analyses on the relation between handedness and effective connectivity in the core face perception network for the conjunction [F-O] ∩ [F-S].

First, random effects Bayesian model selection (BMS) at the family level again selected different winning families for right- and left-handers. For right-handers, family B was the most likely family (expected probability: 0.43; exceedance probability: 0.71), whereas, for left-handers, the most likely family was family F (expected probability: 0.68; exceedance probability: 0.99). A between-group comparison of model frequencies however provided no evidence for a difference in model frequencies between the two handedness groups (i.e., posterior probability of the model frequencies being equal for right- and left-handers: *p* > 0.05). These findings are virtually identical with the observations from our main analysis.

Second, we performed Bayesian model averaging (BMA) across all 96 models within the standard Occam’s window to compute individual connectivity parameters (including only models with a posterior odds ratio above *p* > 0.05; see Methods for detailed information). Using a two-sample *t*-test for each parameter, we did not observe significantly different endogenous connectivity parameters between right- and left-handers (Supplementary Table S1). Similarly, right- and left-handers did not significantly differ with regard to their driving input estimates, which is in line with the main analysis (cf. Table 2). For the modulatory parameters, we found significant differences between right- and left-handers (Supplementary Table S2). In line with our main analysis (cf. Table 3), left-handers showed enhanced modulations by RVF on the intrahemispheric forward connection from left EVC to left FFA and the interhemispheric connection from left to right FFA. Additionally, for left-handers, the intrahemispheric forward connection from left EVC to left FFA and the interhemispheric connection from right to left FFA were significantly stronger modulated by face processing.

It is important to note, however, that none of the handedness-related differences actually survived FDR correction. Hence, the effects tend to be slightly diminished as compared to the main analysis. This is not surprising given the loss in explanatory power attributable to the exclusion of six subjects (3 right-handers and 3 left-handers) when defining the ROIs from the conjunction analysis. Having said this, from a qualitative point of view, the effective connectivity results are highly consistent across the two analyses (i.e., [2*F]-[O+S] vs. [F-O] ∩ [F-S]).

**Supplementary Figure S1**

**
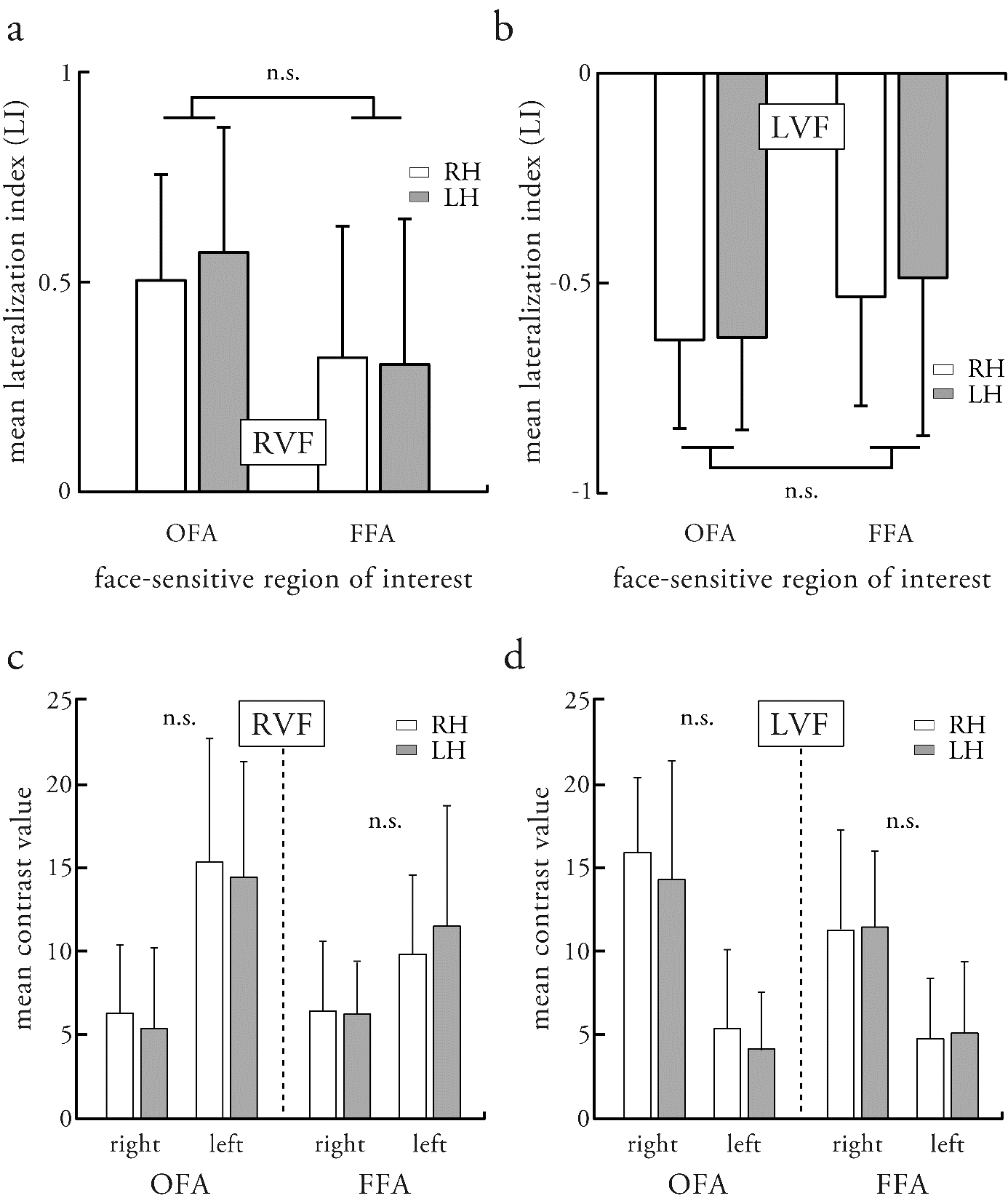
**

**Figure S1:** Specificity of handedness-related differences in cerebral lateralization of the core face perception network. **(a)** Mean and standard deviation of the lateralization indices (LI) of BOLD activity in OFA and FFA for right-handers (white) and left-handers (gray) for the right visual field (RVF) contrast; **(b)** as well as for the left visual field (LVF) contrast. The n.s. illustrates that there was no significant handedness×region interaction. **(c)** Mean and standard deviation of the mean contrast value in all four face-sensitive regions for right-handers (white) and left-handers (gray) for the RVF contrast; **(d)** as well as for the LVF contrast. The n.s. illustrates that there was no significant handedness×hemisphere interaction. Overall, these results suggest that right- and left-handers showed similar hemispheric lateralization of BOLD activity (to the visual field baseline contrasts) in the OFA, as well as in the FFA.

**Supplementary Figure S2**

**
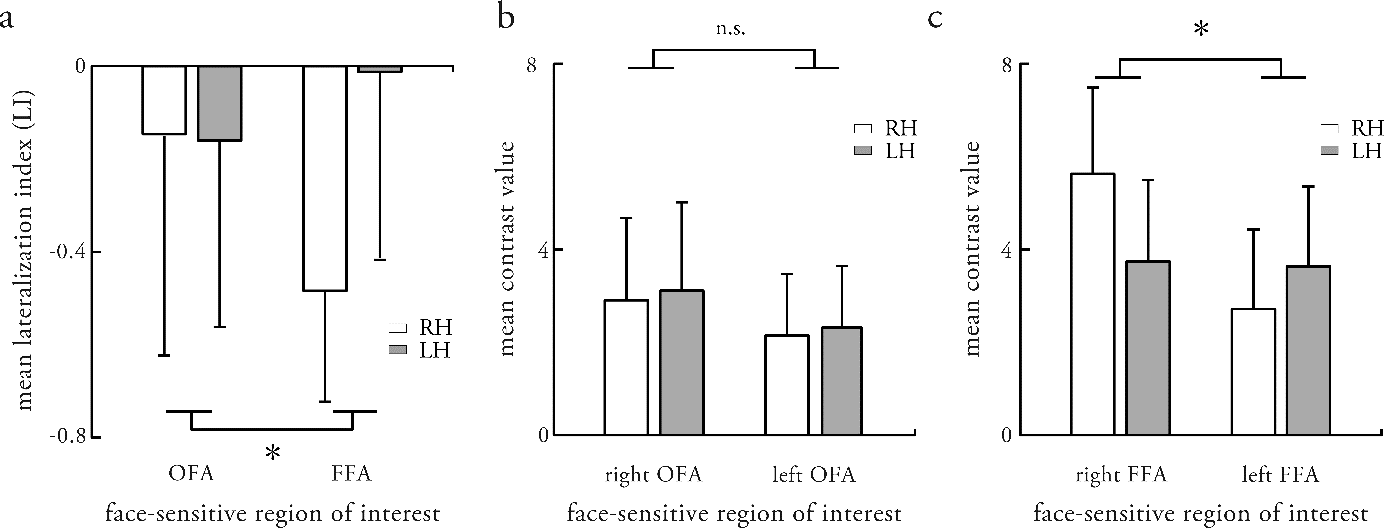
**

**Figure S2:** Cerebral lateralization of the core face perception network in right- and left-handers for the conjunction analysis ([F-O] ∩ [F-S]). **(a)** Mean and standard deviation of the lateralization indices (LI) of BOLD activity in OFA and FFA for right-handers (white) and left-handers (gray). The * denotes a significant handedness×region interaction, suggesting that right- and left-handers showed differential hemispheric lateralization of BOLD activity in the FFA, but not in the OFA. **(b)** Mean and standard deviation of the mean contrast value in the OFA for right-handers (white) and left-handers (gray). The n.s. illustrates that there was no significant handedness×hemisphere interaction, suggesting that right- and left-handers showed similar hemispheric lateralization of BOLD activity in the OFA. **(c)** Mean and standard deviation of the mean contrast value in the FFA for right-handers (white) and left-handers (gray). The * denotes a significant handedness×hemisphere interaction, suggesting that right- and left-handers showed differential hemispheric lateralization of BOLD activity in the FFA.

**Supplementary Figure S3**

**
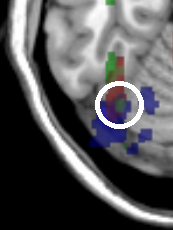
**

**Figure S3:** Overlay of VBM and fMRI results. Clusters show handedness-related differences in gray matter volume (blue cluster), as well as face-sensitive activation in right-handers (red cluster) and left-handers (green cluster). The face-sensitive activations represent the group-level activation patterns (*p* < 0.001, uncorrected). This illustrates that there is some degree of overlap in the fusiform gyrus between the anatomical differences between right- and left-handers and their face-sensitive BOLD activation (white circle).

**Supplementary Figure S4**

**
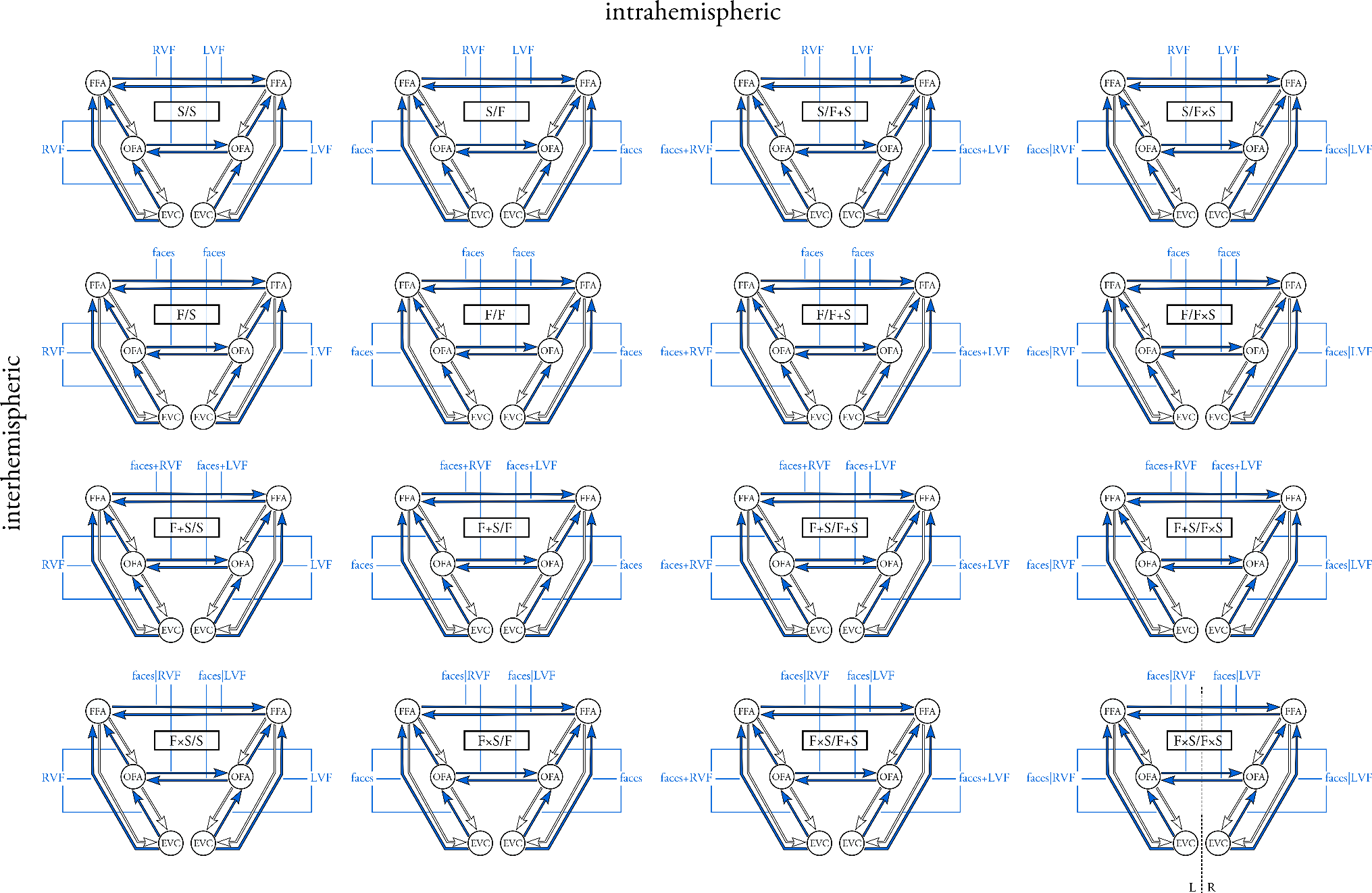
**

**Figure S4:** Structure of all models within the family F, implementing different hypotheses on the effective connectivity in the face perception network. As the visual stimuli were presented in the periphery, RVF and LVF were the driving inputs to the contralateral EVC (not shown here). Forward and backward intrahemispheric endogenous connections were set between V1 and OFA, between V1 and FFA, and between OFA and FFA. Additionally, reciprocal interhemispheric connections were set between bilateral OFA, and between bilateral FFA, but not between bilateral V1. Arrows indicate the presence and directionality of the endogenous connections. Whereas endogenous connectivity and driving inputs were the same for all models, modulatory influences differed. Connections were either modulated by 1) the visual field (S), 2) face perception (F), 3) face perception and visual field (F+S), or 4) face perception, but only when the stimuli were presented in the respective visual field (F×S). All four possibilities exist for intra- and interhemispheric connections, hence, systematically varying all combinations resulted in 16 distinct models. Endogenous connections that were modulated are colored in blue. Models are named by first listing the type of interhemispheric modulation, followed by the type of intrahemispheric modulation. LVF = left visual field; RVF = right visual field; faces = face perception; L = left hemisphere; R = right hemisphere

**Supplementary Table S1:** Endogenous connectivity parameter estimates for right- and left-handers for the conjunction analysis ([F-O] ∩ [F-S]).

|  | right-handers (RH) | | left-handers (LH) | | Difference (RH vs. LH) |
| --- | --- | --- | --- | --- | --- |
|  | mean ± std | *p* | mean ± std | *p* | *p* |
| FFA_L 🡪 FFA_R | **0.271 ± 0.149** | **1.29e-6** | **0.240 ± 0.072** | **3.28e-10** | 4.49e-1 |
| FFA_L 🡪 OFA_L | **0.142 ± 0.116** | **1.22e-4** | **0.148 ± 0.092** | **5.19e-6** | 8.63e-1 |
| FFA_L 🡪 EVC_L | -0.085 ± 0.252 | 1.82e-1 | -0.090 ± 0.179 | 5.38e-2 | 9.46e-1 |
| FFA_R 🡪 FFA_L | **0.234 ± 0.153** | **1.04e-5** | **0.223 ± 0.085** | **8.89e-9** | 8.09e-1 |
| FFA_R 🡪 OFA_R | **0.172 ± 0.176** | **9.77e-4** | **0.189 ± 0.141** | **4.45e-5** | 7.45e-1 |
| FFA_R 🡪 EVC_R | -0.036 ± 0.208 | 4.91e-1 | -0.063 ± 0.230 | 2.75e-1 | 7.18e-1 |
| OFA_L 🡪 OFA_R | **0.178 ± 0.122** | **1.91e-5** | **0.157 ± 0.152** | **5.94e-4** | 6.70e-1 |
| OFA_L 🡪 FFA_L | **0.141 ± 0.131** | **4.02e-4** | 0.055 ± 0.125 | 9.95e-2 | 5.38e-2 |
| OFA_L 🡪 EVC_L | **-0.201 ± 0.153** | **5.71e-5** | **-0.135 ± 0.218** | **2.14e-2** | 3.10e-1 |
| OFA_R 🡪 OFA_L | **0.176 ± 0.084** | **2.21e-7** | **0.180 ± 0.152** | **1.70e-4** | 9.18e-1 |
| OFA_R 🡪 FFA_R | **0.164 ± 0.156** | **5.25e-4** | **0.112 ± 0.079** | **2.46e-5** | 2.34e-1 |
| OFA_R 🡪 EVC_R | -0.083 ± 0.212 | 1.27e-1 | -0.025 ± 0.260 | 6.92e-1 | 4.85e-1 |
| EVC_L 🡪 FFA_L | **0.105 ± 0.124** | **3.10e-3** | **0.127 ± 0.053** | **3.46e-8** | 5.19e-1 |
| EVC_L 🡪 OFA_L | **0.165 ± 0.124** | **4.96e-5** | **0.205 ± 0.100** | **2.62e-7** | 3.07e-1 |
| EVC_R 🡪 FFA_R | **0.117 ± 0.085** | **3.50e-5** | **0.136 ± 0.077** | **1.91e-6** | 5.16e-1 |
| EVC_R 🡪 OFA_R | **0.149 ± 0.091** | **4.76e-6** | **0.181 ± 0.091** | **4.19e-7** | 3.12e-1 |

*Note.* Endogenous parameter estimates are provided in terms of their mean and standard deviation (i.e., mean ± std), and were estimated using BMA over all models within the standard Occam’s window (*p* < 0.05). Significant connections at the group level (*p* < 0.05, FDR-corrected) are printed bold (*columns left of dashed line*). Additionally, for each parameter, the uncorrected p-value of the two-sample *t*-tests on handedness-related differences in the endogenous parameter estimates is provided. Connections showing a significant handedness-related difference (*p* < 0.05, uncorrected) are printed bold (*columns right of dashed line*). RH = right-handers, LH = left-handers.

**Supplementary Table S2:** Modulatory parameter estimates for right- and left-handers for the conjunction analysis ([F-O] ∩ [F-S]).

|  | right-handers (RH) | | left-handers (LH) | | difference  (RH vs. LH) |
| --- | --- | --- | --- | --- | --- |
|  | mean ± std | *p* | mean ± std | *p* | *p* |
| *modulatory parameters (RVF)* | | | | |  |
| FFA_L 🡪 FFA_R | **0.105 ± 0.117** | **1.90e-3** | **0.177 ± 0.061** | **1.98e-9** | **3.13e-2** |
| OFA_L 🡪 OFA_R | 0.042 ± 0.196 | 3.91e-1 | -0.003 ± 0.130 | 9.15e-1 | 4.33e-1 |
| OFA_L 🡪 FFA_L | -0.047 ± 0.096 | 6.16e-2 | **-0.087 ± 0.114** | **5.90e-3** | 2.65e-1 |
| EVC_L 🡪 FFA_L | -0.003 ± 0.042 | 1.67e-1 | **0.052 ± 0.051** | **6.80e-4** | **1.80e-3** |
| EVC_L 🡪 OFA_L | **0.068 ± 0.081** | **3.30e-3** | **0.079 ± 0.121** | **1.64e-2** | 7.62e-1 |
| *modulatory parameters (LVF)* | | | | |  |
| FFA_R 🡪 FFA_L | **0.093 ± 0.138** | **1.32e-2** | **0.148 ± 0.075** | **2.42e-6** | 1.64e-1 |
| OFA_R 🡪 OFA_L | **0.105 ± 0.122** | **2.60e-3** | 0.060 ± 0.127 | 6.90e-2 | 3.03e-1 |
| OFA_R 🡪 FFA_R | **-0.094 ± 0.116** | **4.30e-3** | **-0.062 ± 0.090** | **1.16e-2** | 3.90e-1 |
| EVC_R 🡪 FFA_R | -0.005 ± 0.025 | 3.90e-1 | -0.011 ± 0.065 | 4.95e-1 | 7.39e-1 |
| EVC_R 🡪 OFA_R | **0.068 ± 0.095** | **9.10e-3** | 0.069 ± 0.124 | 3.50e-2 | 9.71e-1 |
| *modulatory parameters (faces)* | | | | |  |
| FFA_L 🡪 FFA_R | **0.172 ± 0.073** | **4.08e-8** | **0.156 ± 0.068** | **5.45e-8** | 5.09e-1 |
| FFA_R 🡪 FFA_L | **0.053 ± 0.093** | **3.09e-2** | **0.129 ± 0.077** | **3.37e-6** | **1.40e-2** |
| OFA_L 🡪 OFA_R | **0.154 ± 0.132** | **1.94e-4** | **0.188 ± 0.096** | **5.18e-7** | 4.06e-1 |
| OFA_L 🡪 FFA_L | **0.069 ± 0.088** | **5.40e-3** | **0.063 ± 0.080** | **5.20e-3** | 8.33e-1 |
| OFA_R 🡪 OFA_L | **0.099 ± 0.051** | **5.40e-7** | **0.111 ± 0.061** | **1.14e-6** | 5.21e-1 |
| OFA_R 🡪 FFA_R | **0.110 ± 0.101** | **3.72e-4** | **0.077 ± 0.089** | **2.70e-3** | 3.19e-2 |
| EVC_L 🡪 FFA_L | **0.066 ± 0.078** | **3.10e-3** | **0.144 ± 0.068** | **1.74e-7** | **3.50e-3** |
| EVC_L 🡪 OFA_L | **0.126 ± 0.134** | **1.10e-3** | **0.133 ± 0.079** | **3.59e-6** | 8.62e-1 |
| EVC_R 🡪 FFA_R | **0.067 ± 0.090** | **7.30e-3** | **0.109 ± 0.064** | **2.59e-6** | 1.25e-1 |
| EVC_R 🡪 OFA_R | **0.083 ± 0.087** | **1.20e-3** | **0.132 ± 0.108** | **1.24e-4** | 1.57e-1 |
| *modulatory parameters (faces|RVF)* | | | |  |  |
| FFA_L 🡪 FFA_R | - | - | 0.000 ± 0.001 | 3.32e-1 | 3.25e-1 |
| OFA_L 🡪 OFA_R | - | - | 0.000 ± 0.001 | 3.32e-1 | 3.25e-1 |
| OFA_L 🡪 FFA_L | -0.000 ± 0.006 | 8.03e-2 | -0.000 ± 0.001 | 3.81e-1 | 8.62e-1 |
| EVC_L 🡪 FFA_L | **0.019 ± 0.025** | **6.80e-3** | 0.004 ± 0.009 | 1.00e-1 | **2.60e-2** |
| EVC_L 🡪 OFA_L | - | - | 0.001 ± 0.002 | 1.57e-1 | 1.47e-1 |
| *modulatory parameters (faces|LVF)* | | | | |  |
| FFA_R 🡪 FFA_L | - | - | 0.001 ± 0.004 | 3.32e-1 | 3.25e-1 |
| OFA_R 🡪 OFA_L | - | - | 0.001 ± 0.003 | 3.32e-1 | 3.25e-1 |
| OFA_R 🡪 FFA_R | 0.000 ± 0.004 | 8.87e-1 | 0.000 ± 0.004 | 8.56e-1 | 9.82e-1 |
| EVC_R 🡪 FFA_R | **0.020 ± 0.027** | **8.60e-3** | 0.006 ± 0.017 | 2.02e-1 | 7.55e-2 |
| EVC_R 🡪 OFA_R | - | - | 0.001 ± 0.004 | 2.47e-1 | 2.39e-1 |

*Note.* Modulatory parameter estimates are provided in terms of their mean and standard deviation (i.e., mean ± std), and were estimated using BMA over all models within the standard Occam’s window (*p* < 0.05). Significant modulatory influences at the group level (*p* < 0.05, FDR-corrected) are printed bold (*columns left of dashed line*). Additionally, for each parameter, the uncorrected p-value of the two-sample *t*-tests on handedness-related differences in the modulatory parameter estimates is provided. Modulatory influences showing a significant handedness-related difference (*p* < 0.05, uncorrected) are printed bold (*columns right of dashed line*). RH = right-handers, LH = left-handers.
